# Role of prokinetic drugs prior to endoscopy in acute upper gastrointestinal bleeding: a systematic review and meta-analysis

**DOI:** 10.3389/fgstr.2026.1930145

**Published:** 2026-09-16

**Authors:** Mohamad Yousef Alabdallat, Tarek Bakain, Abdulrahman Atasi, Omar A. Oudit, Mohammad Ghannam, Zaid AlRashid, Khaled Moghib, Emeka Stanley Obi, Muhammad Sarfaraz, Chidiebele Emmanuel Omaliko, Derrick Cheung

**Affiliations:** 1 Brookdale University Hospital Medical Center, Brooklyn, NY, United States; 2 Washington University, St. Louis, MO, United States; 3 Faculty of Medicine, Kasr Al-Ainy, Cairo University, Cairo, Egypt

**Keywords:** endoscopy, erythromycin, metoclopramide, prokinetic agents, upper gastrointestinal bleeding

## Abstract

**Background:**

Acute upper gastrointestinal bleeding (UGIB) is a common gastrointestinal emergency in which retained blood and clots may impair endoscopic visualization, delay diagnosis, and increase the need for repeat endoscopy. Pre-endoscopic prokinetic therapy, particularly erythromycin, may improve gastric clearance before endoscopy, but its effects on clinical outcomes remain uncertain.

**Objective:**

To evaluate the efficacy and safety of pre-endoscopic prokinetic therapy in adults with acute UGIB, with attention to the strength of evidence for erythromycin and metoclopramide.

**Methods:**

We conducted a systematic review and meta-analysis of randomized controlled trials evaluating pre-endoscopic prokinetic therapy in adults with acute UGIB. The primary outcome was adequate gastric emptying or adequate endoscopic visualization. Secondary outcomes included repeat endoscopy, mortality, endoscopy duration, blood transfusion requirement, length of hospital stay, endoscopic visualization score, bleeding-source identification, rebleeding, and adverse events. Dichotomous outcomes were pooled as odds ratios (ORs), and continuous outcomes as mean differences (MDs). Because visualization scores were measured using different scales, this outcome was pooled using standardized mean difference (SMD). Certainty of evidence was assessed using GRADE.

**Results:**

Ten randomized trials including 1,077 participants were included. Eight trials evaluated erythromycin and two evaluated metoclopramide. Pre-endoscopic prokinetic therapy improved adequate gastric emptying or endoscopic visualization (OR 3.68, 95% CI 2.06–6.60; p < 0.0001; I^2^ = 59.9%) and reduced repeat endoscopy (OR 0.54, 95% CI 0.37–0.79; p = 0.0015; I^2^ = 20.8%). Continuous endoscopic visualization scores also favored prokinetic therapy when analyzed using SMD (SMD 0.47, 95% CI 0.21–0.72; p = 0.0003; I^2^ = 62.1%). No clear benefit was demonstrated for mortality (OR 0.59, 95% CI 0.32–1.09), rebleeding (OR 1.12, 95% CI 0.64–1.97), blood transfusion requirement (MD −0.64 units, 95% CI −1.44 to 0.15), or endoscopy duration (MD −1.67 minutes, 95% CI −5.59 to 2.25) under random-effects models. Length of hospital stay was shorter with prokinetic therapy (MD −1.30 days, 95% CI −2.11 to −0.49), although this outcome may be influenced by healthcare-system factors. Safety reporting was limited and inconsistent; no consistent serious safety signal was identified, but the trials were not powered to detect uncommon adverse events such as QT prolongation, arrhythmia, or extrapyramidal reactions.

**Conclusion:**

Pre-endoscopic prokinetic therapy, supported mainly by erythromycin-based randomized evidence, improves gastric emptying/endoscopic visualization and probably reduces repeat endoscopy in adults with acute UGIB. These findings should be interpreted primarily as procedural benefits rather than evidence of improved hard clinical outcomes. Evidence for metoclopramide remains limited and less consistent, and current data do not support a definitive class effect for all prokinetic drugs.

## 1 Introduction

Acute upper gastrointestinal bleeding (UGIB) is a common and potentially life-threatening gastrointestinal emergency associated with substantial morbidity, mortality, and healthcare burden. Early risk stratification, hemodynamic resuscitation, and timely upper gastrointestinal endoscopy are central to management (1, 2). Current guidelines recommend early endoscopy, generally within 24 hours after presentation or after adequate resuscitation, because endoscopy enables diagnosis, risk stratification, and definitive therapy when indicated (1–4).

The effectiveness of endoscopy in acute UGIB depends heavily on adequate visualization of the gastric and duodenal mucosa. However, retained blood, clots, and gastric contents can obscure the bleeding source, reduce diagnostic accuracy, prolong the procedure, and increase the need for incomplete or repeat endoscopy. Therefore, strategies that improve gastric clearance before endoscopy may enhance the diagnostic and therapeutic yield of the index procedure (3, 4).

Pre-endoscopic prokinetic therapy has been proposed as a simple pharmacologic strategy to improve gastric emptying and mucosal visualization before endoscopy. Erythromycin, a motilin receptor agonist, promotes gastric contractions and accelerates gastric emptying, whereas metoclopramide has prokinetic activity mainly through dopamine D2 receptor antagonism and serotonergic effects (5, 6). Current guidelines support intravenous erythromycin before endoscopy in selected patients with clinically severe or ongoing active UGIB, but the evidence for metoclopramide remains less certain (3, 4).

Previous trials and evidence syntheses suggest that erythromycin may improve gastric mucosal visualization and reduce repeat endoscopy, but important uncertainties remain. Earlier meta-analyses were limited by small sample sizes, heterogeneous outcome definitions, and variable comparator groups, including placebo, standard care, gastric lavage, and combinations of prokinetic therapy with lavage (5, 7–9). In addition, most earlier evidence focused on erythromycin, while direct evidence regarding metoclopramide was limited.

More recent randomized trials and network meta-analyses have renewed interest in this topic, particularly regarding the comparative effects of erythromycin, metoclopramide, and gastric lavage (6, 10–12). However, conclusions remain influenced by limited head-to-head evidence, heterogeneity across trial designs, and inconsistent effects on clinically important outcomes such as repeat endoscopy, rebleeding, hospital stay, and mortality. Therefore, this systematic review and meta-analysis aim to provide an updated evaluation of the efficacy and safety of prokinetic drugs administered before endoscopy in adults with acute UGIB.

## 2 Methods

This study was conducted following the Cochrane Handbook for Systematic Reviews of Interventions (13). The results were reported as specified by the Preferred Reporting Items for Systematic Reviews and Meta-Analyses (PRISMA) statement (14). Additionally, this study has been registered with PROSPERO under the identification number (CRD420261412891).

### 2.1 Information sources and search strategy

A comprehensive literature search was conducted in PubMed/MEDLINE, Scopus, Web of Science, Cochrane Library/CENTRAL, and Embase from database inception to 30 June 2026. The search strategy combined controlled vocabulary and free-text terms related to acute upper gastrointestinal bleeding, prokinetic agents, erythromycin, metoclopramide, gastric emptying, and upper gastrointestinal endoscopy. No restrictions were applied regarding publication year. The detailed search strategy for each database is provided in **Appendix Table A1**. Two reviewers independently performed the search and screened the retrieved records. Inter-rater agreement was assessed using Cohen’s kappa when applicable. Disagreements during title/abstract screening, full-text review, data extraction, and risk-of-bias assessment were resolved by discussion, with consultation of a third reviewer when consensus was not reached. A completed PRISMA 2020 checklist is provided as Supplementary File. Specific reasons for full-text exclusion are provided in the PRISMA flow diagram.

### 2.2 Eligibility criteria

#### 2.2.1 Inclusion criteria

Studies were selected according to the PICOS framework.

- Populations: Eligible populations included adults aged 18 years or older presenting with acute upper gastrointestinal bleeding and undergoing upper gastrointestinal endoscopy. Studies including patients with variceal, non-variceal, or mixed causes of upper gastrointestinal bleeding were eligible.
- Intervention: The intervention of interest was administration of a prokinetic drug before endoscopy, including erythromycin, metoclopramide, or other prokinetic agents, regardless of dose, route, timing, or duration. Studies in which prokinetic therapy was combined with gastric lavage were also eligible if a relevant comparator group was available.
- Comparator: Eligible comparators included placebo, no prokinetic therapy, standard care without prokinetic therapy, gastric lavage alone, or placebo combined with gastric lavage.
- Outcomes: Eligible studies were required to report at least one relevant outcome, including adequate gastric emptying or adequate endoscopic mucosal visualization, visualization score, need for second-look or repeat endoscopy, all-cause mortality, endoscopy duration, identification of the bleeding source, need for endoscopic therapy, successful hemostasis, blood transfusion requirement, rebleeding, length of hospital stay, adverse events, or other reported safety outcomes.
- Study types: Randomized controlled trials were eligible for inclusion. Quasi-randomized trials were considered eligible if allocation was clearly described and sufficient outcome data were available.

#### 2.2.2 Exclusion criteria

Studies were excluded if they met any of the following criteria: non-human or *in vitro* studies; pediatric studies; observational studies; case reports; case series; reviews; editorials; commentaries; letters without original data; conference abstracts without sufficient extractable data; studies not involving acute upper gastrointestinal bleeding; studies not involving upper gastrointestinal endoscopy; studies without pre-endoscopic administration of a prokinetic agent; studies without an eligible comparator group; studies reporting no relevant outcomes; duplicate or overlapping populations unless additional non-duplicative outcome data were available; or studies with insufficient data for extraction after full-text assessment.

### 2.3 Study selection and data management

All retrieved records were exported to reference-management software, and duplicate records were removed before screening. The screening process was conducted in two phases. First, two reviewers independently screened titles and abstracts to identify potentially eligible studies. Second, the full texts of potentially relevant articles were reviewed independently by the same two reviewers to confirm eligibility. Rayyan software was used to facilitate the screening process. Disagreements were resolved through discussion, and unresolved conflicts were adjudicated by a third reviewer (15).

### 2.4 Data extraction

Data extraction was performed independently by two reviewers using a standardized data extraction form. Any discrepancies were resolved through discussion and, when necessary, consultation with a third reviewer. For each included study, the following information was extracted: first author, year of publication, country, study design, study setting, sample size, number of participants in each treatment arm, eligibility criteria, baseline participant characteristics, type and cause of upper gastrointestinal bleeding, intervention details, comparator details, use of gastric lavage, timing of drug administration before endoscopy, dose, route of administration, and timing of endoscopy. The primary outcome extracted was adequate gastric emptying before endoscopy, operationally defined as adequate endoscopic visualization, clear stomach, empty stomach, satisfactory visualization, or good-quality endoscopic visualization according to the definitions used by the individual trials. When available, visualization scores were also extracted as continuous outcomes. Secondary outcomes included need for second-look or repeat endoscopy, all-cause mortality, endoscopy duration, identification of the bleeding source, need for endoscopic therapy, successful hemostasis, blood transfusion requirement, rebleeding, length of hospital stay, and adverse events or complications related to prokinetic therapy, gastric lavage, or endoscopy. For repeat endoscopy, rebleeding, and mortality, the reported time point was recorded for each study. For dichotomous outcomes, the number of events and the total number of participants in each group were extracted. For continuous outcomes, means and standard deviations were extracted when available. If studies reported medians with ranges or interquartile ranges, these were extracted as reported and converted to means and standard deviations when appropriate using established statistical methods. When outcome data were incomplete, unclear, or reported only graphically, the available information was extracted as accurately as possible, and the issue was documented. When multiple publications reported overlapping data, the most complete and relevant dataset was used.

### 2.5 Risk of bias assessment

Two reviewers independently assessed the risk of bias of included studies. Randomized controlled trials were evaluated using the revised Cochrane Risk of Bias tool for randomized trials (RoB 2). The assessed domains included bias arising from the randomization process, bias due to deviations from intended interventions, bias due to missing outcome data, bias in outcome measurement, and bias in selection of the reported result. Each domain was judged as low risk, some concerns, or high risk of bias, and an overall judgment was assigned for each randomized trial (16).

Disagreements between reviewers were resolved by discussion or by consultation with a third reviewer. Risk-of-bias figures were generated using the robvis visualization tool (17).

Because the primary outcome involved subjective assessment of endoscopic visualization, particular attention was given to RoB 2 Domain 4, bias in measurement of the outcome. Specifically, we re-assessed whether the endoscopist or outcome assessor who judged mucosal visualization was blinded to treatment allocation. Studies with clearly blinded endoscopists or blinded outcome assessors were judged as low risk for Domain 4, whereas studies with unclear assessor blinding were judged as having some concerns for Domain 4 unless objective outcome measurement or other safeguards reduced the likelihood of bias.

### 2.6 Publication bias

Publication bias and small-study effects were planned to be assessed using funnel plot inspection and Egger’s test when at least 10 comparisons were available for an outcome. For outcomes with fewer than 10 comparisons, formal tests for funnel plot asymmetry were not performed because of limited statistical power (18).

### 2.7 Choice of the meta-analysis model

Meta-analyses were performed using both common-effect and random-effects models, with the random-effects model considered the primary model for interpretation. This choice was made because clinical and methodological heterogeneity was expected across the included trials, including differences in the prokinetic agent used, route and dose of administration, timing before endoscopy, comparator type, use of gastric lavage, bleeding etiology, and outcome definitions.

For dichotomous outcomes, pooled odds ratios (ORs) with 95% confidence intervals (CIs) were calculated. The Mantel–Haenszel method was used for common-effect analyses, while inverse-variance random-effects models were used for random-effects analyses. For continuous outcomes, pooled mean differences (MDs) with 95% CIs were calculated when outcomes were measured on the same scale. When studies reported medians with interquartile ranges, values were converted to means and standard deviations using the method described by Wan et al. when appropriate.

Between-study variance was estimated using the restricted maximum-likelihood (REML) estimator. Statistical heterogeneity was assessed using Cochran’s Q test and quantified using the I^2^ statistic, with higher I^2^ values indicating greater between-study inconsistency. Because random-effects models account for both within-study and between-study variability, they were preferred when heterogeneity was present or when variation in study design was clinically expected. Common-effect estimates were also reported as sensitivity or supportive analyses.

For multi-arm randomized trials with more than one eligible intervention arm sharing the same comparator group, the shared comparator group was split to avoid double-counting participants in pairwise meta-analyses. For dichotomous outcomes, both the number of events and the total number of participants in the shared comparator group were divided proportionally across the eligible comparisons. For continuous outcomes, the comparator sample size was divided across comparisons, while the mean and standard deviation were retained unchanged, as recommended in the Cochrane Handbook. This approach preserves independence between pairwise comparisons while allowing all relevant intervention arms to contribute to the analysis. To assess whether this method influenced the pooled estimates, sensitivity analyses were conducted by excluding multi-arm comparisons or by retaining only one eligible comparison from each multi-arm trial when feasible (19–23).

### 2.8 Calculation of missing data

When continuous outcomes were reported as medians with interquartile ranges or ranges, means and standard deviations were estimated using the method described by Wan et al. when appropriate. This conversion was applied only when the outcome was considered suitable for quantitative synthesis and when the reported median, range, and/or interquartile range did not suggest marked skewness. The assumption underlying these conversions is that the distribution is approximately symmetric; therefore, converted values were interpreted cautiously, particularly for outcomes with clinical or statistical heterogeneity. When standard deviations were not directly reported but standard errors were available, standard deviations were calculated using the formula SD = SE × √n. No change-score outcomes were pooled in this meta-analysis, and no SD-change imputation was used. Outcomes with insufficient, unclear, or highly skewed data were summarized narratively and excluded from quantitative synthesis for that specific analysis. Sensitivity analyses were conducted, when feasible, by excluding studies with converted continuous data to assess the robustness of the pooled estimates (22, 24, 25, 26).

### 2.9 Statistical analysis

Statistical analyses were performed using R software. Forest plots were generated to display individual study estimates and pooled effect estimates. For dichotomous outcomes, pooled odds ratios (ORs) with 95% confidence intervals (CIs) were calculated. The Mantel–Haenszel method was used for common-effect analyses, and inverse-variance random-effects models were used for random-effects analyses. For continuous outcomes, pooled mean differences (MDs) with 95% CIs were calculated when outcomes were reported using comparable units, such as endoscopy duration, blood transfusion requirement, and length of hospital stay.

When continuous outcomes were reported as medians with interquartile ranges, means and standard deviations were estimated using the method described by Wan et al. when appropriate. For multi-arm trials with a shared comparator group, the comparator group was split across relevant comparisons to avoid double-counting. For dichotomous outcomes, comparator events and totals were divided proportionally; for continuous outcomes, the comparator sample size was divided, while the mean and standard deviation were retained.

The random-effects model was considered the primary model for interpretation because clinical and methodological heterogeneity was expected across studies, including differences in prokinetic agent, dose, route of administration, timing before endoscopy, comparator type, use of gastric lavage, and outcome definition. Common-effect estimates were also reported as supportive analyses. A two-sided p-value <0.05 was considered statistically significant, except for Cochran’s Q test for heterogeneity, where p <0.10 was considered suggestive of statistically significant heterogeneity (19).

For continuous outcomes measured using different scoring systems or scales across studies, standardized mean differences (SMDs) with 95% confidence intervals were calculated using Hedges’ g correction. This approach was applied to the endoscopic visualization score outcome because included trials used different scales, including 0–3, 0–8, 0–10, and 0–16 scoring systems.

### 2.10 Assessment of heterogeneity

Statistical heterogeneity was assessed using Cochran’s Q test and quantified using the I^2^ statistic. A Cochran’s Q test p-value < 0.10 was considered suggestive of statistically significant heterogeneity. I^2^ values were interpreted according to Cochrane guidance: 0%–40% may represent low or unimportant heterogeneity, 30%–60% may represent moderate heterogeneity, 50%–90% may represent substantial heterogeneity, and 75%–100% may represent considerable heterogeneity.

Potential sources of heterogeneity were explored through subgroup and sensitivity analyses when sufficient data were available. Prespecified subgroup analyses included type of prokinetic agent, mainly erythromycin versus metoclopramide, and comparator strategy, particularly studies with gastric lavage versus those without gastric lavage. Additional exploration considered differences in route of administration, bleeding etiology, and outcome definition when clinically relevant.

Sensitivity analyses were performed using leave-one-out analysis to evaluate whether pooled estimates were driven by any single study. Where applicable, sensitivity analyses also considered alternative outcome definitions, such as clinically indicated repeat endoscopy versus any repeat endoscopy, and the impact of excluding studies with methodological concerns or outcomes reported in non-standard formats (27).

## 3 Results

### 3.1 Search and screening

The electronic database search was performed on 30 June 2026. A total of 633 records were identified across five databases: PubMed/MEDLINE (n = 134), Scopus (n = 304), Web of Science (n = 56), Cochrane Library (n = 89), and Embase (n = 50). After the removal of 208 duplicate records, 425 unique records remained for title and abstract screening. Following title and abstract screening, 24 reports were retrieved for full-text assessment. Of these, 10 randomized controlled trials met the eligibility criteria and were included in the final systematic review and meta-analysis (6, 12, 28–35). The excluded full-text articles did not meet the eligibility criteria because they were not randomized controlled trials, did not include an eligible comparator, did not evaluate pre-endoscopic prokinetic therapy, were abstract-only reports with insufficient extractable data, or represented duplicate/overlapping evidence (**Figure 1**).

**Figure 1:**
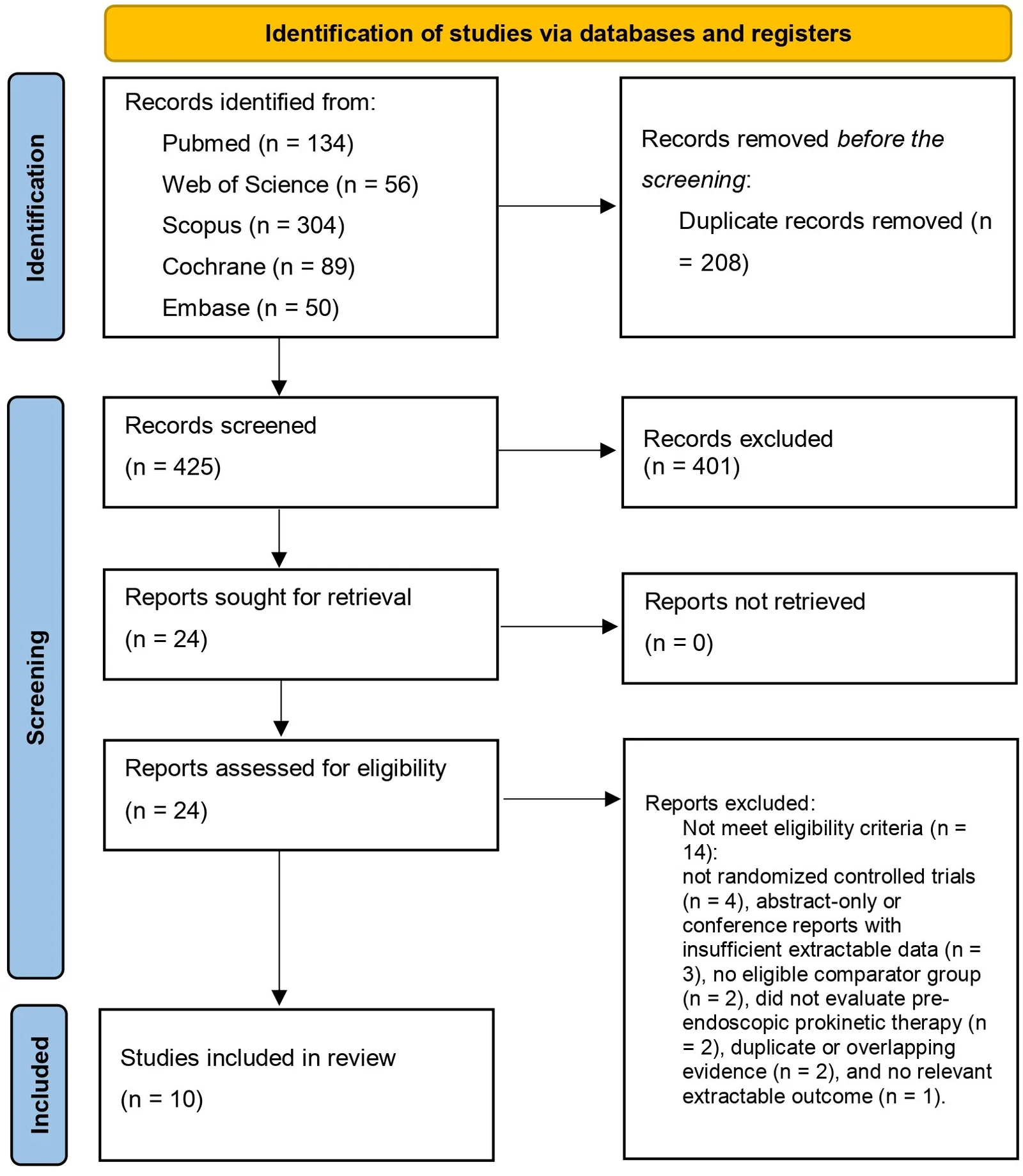
PRISMA flow diagram.

### 3.2 Study characteristics

The 10 included randomized controlled trials were published between 2002 and 2024 and evaluated the role of prokinetic therapy before upper gastrointestinal endoscopy in adults with acute upper gastrointestinal bleeding. The trials were conducted in different geographic settings, including France, Switzerland, Saudi Arabia, Iran, Korea, Pakistan, and Thailand. Most studies were single-center trials, while two were multicenter studies: Pateron et al. included six emergency departments in France, and Manupeeraphant et al. (6) was conducted across multiple centers in Thailand.

The included studies enrolled a total of 1,077 participants. Sample sizes ranged from 40 participants in Javad Ehsani Ardakani et al. (30) to 284 analyzed participants in Manupeeraphant et al. (6) Most trials included patients presenting with hematemesis, melena, or clinical evidence of acute upper gastrointestinal bleeding within 12 hours, although Manupeeraphant et al. (6) included patients undergoing EGD within 24 hours after diagnosis. Several studies included mixed variceal and non-variceal bleeding populations, whereas Altraif et al. (29) focused on patients with confirmed variceal bleeding, and Na et al. (34) included only non-variceal upper gastrointestinal bleeding.

Erythromycin was the most commonly evaluated prokinetic agent and was used in eight trials. Most erythromycin trials administered the drug intravenously before endoscopy, usually at a dose of 250 mg or 3 mg/kg, with timing ranging from approximately 20 to 90 minutes before EGD. Ali Shah et al. (28) was the only trial evaluating oral erythromycin, using a 500 mg oral suspension 3 hours before endoscopy. Two recent trials evaluated intravenous metoclopramide 10 mg before endoscopy: Manupeeraphant et al. (6) administered metoclopramide 10–30 minutes before EGD, while Vimonsuntirungsri et al. (12) administered metoclopramide 30–120 minutes before EGD.

Comparator groups varied across trials. Several studies used a placebo or no prokinetic therapy, while others incorporated gastric lavage as either a comparator or a co-intervention. Carbonell et al. (31) and Javad Ehsani Ardakani et al. (30) compared erythromycin plus gastric lavage with placebo plus gastric lavage. Pateron et al. (35) and Na et al. (34) included three-arm comparisons involving erythromycin alone, gastric lavage alone, and erythromycin plus gastric lavage. This variation in comparator design was considered clinically important and was addressed in subgroup analyses.

The primary outcome across studies was related to gastric emptying or endoscopic visualization, although definitions differed between trials. Most studies assessed adequate visualization using endoscopic scoring systems, clear stomach definitions, or subjective/objective visualization criteria. Secondary outcomes commonly included need for second-look or repeat endoscopy, endoscopy duration, blood transfusion requirements, identification of the bleeding source, need for endoscopic therapy or hemostasis, rebleeding, length of hospital stay, mortality, and adverse events. (**Tables 1**, **2**).

**Table 1:** Study, design, and intervention characteristics of included trials.

Study	Country/setting	Design	Participants/arms	Intervention	Comparator	Timing before endoscopy	Primary outcome/assessment
Ali Shah et al., 2020 (28)	Pakistan; single center	Single-center randomized placebo-controlled interventional study	60 patients: oral erythromycin 30, placebo 30	Oral erythromycin suspension 500 mg	Oral placebo suspension	3 h before EGD	Endoscopic quality using modified subjective/objective scoring system; total score ≥5/7 defined good-quality endoscopy
Altraif et al., 2011 (29)	Saudi Arabia; tertiary care hospital	Prospective randomized, double-blind, placebo-controlled trial	102 randomized/treated; 90 analyzed with confirmed variceal bleeding: erythromycin 47, placebo 43	IV erythromycin 125 mg in 50 mL normal saline over 10 min	IV placebo normal saline over 10 min	EGD 30 min after completion of infusion	Endoscopic visibility during index endoscopy using modified objective score; procedure duration
Carbonell et al., 2006 (31)	France; intensive care unit	Randomized, controlled, double-blind trial	100 randomized; 99 analyzed: erythromycin 49, placebo 50	Gastric lavage plus IV erythromycin 250 mg in 100 mL isotonic saline over 30 min	Gastric lavage plus IV placebo	EGD 30 min after completion of infusion	Gastric cleansing before endoscopy assessed by subjective and objective criteria
Coffin et al., 2002 (32)	France; ICU/emergency endoscopy setting	Prospective randomized, endoscopist-blinded controlled trial	41 patients: erythromycin 19, control 22	IV erythromycin 3 mg/kg in 125 mL saline over 30 min	No prokinetic treatment/control; standard NG assessment/lavage allowed before randomization	EGD 30–90 min after completion of infusion; equivalent to 60–120 min after infusion initiation	Quality of EGD on 0–3 scale; need for second diagnostic EGD within 8 days
Javad Ehsani Ardakani et al., 2013 (30)	Iran; single center	Prospective randomized, double-blind controlled trial	40 patients: erythromycin 20, placebo 20	NG tube/lavage plus IV erythromycin 3 mg/kg in 100 mL isotonic saline over 5 min	NG tube/lavage plus placebo in 100 mL isotonic saline	EGD 30–60 min after drug/placebo infusion	Endoscopic yield using subjective 0–3 visualization scale and endoscopy duration
Frossard et al., 2002 (33)	Switzerland; single center	Prospective randomized, double-blind, placebo-controlled trial	105 patients: erythromycin 51, placebo 54	IV erythromycin 250 mg in 50 mL isotonic saline over 5 min	IV placebo	EGD 20 min after infusion	Endoscopic yield using subjective and objective scoring systems; endoscopy duration
Manupeeraphant et al., 2024 (6)	Thailand; multicenter	Multicenter randomized, double-blind controlled trial	300 randomized/diagnosed; 284 completed per-protocol analysis: metoclopramide 143, placebo 141	IV metoclopramide 10 mg	IV placebo	10–30 min before EGD	Endoscopic mucosal visualization using modified Avgerinos score; total score range 0–8
Na et al., 2017 (34)	Korea; emergency department, single center	Prospective randomized pilot trial	43 patients: erythromycin 14, gastric lavage 15, erythromycin plus gastric lavage 14	IV erythromycin 250 mg in 50 mL isotonic saline over 30 min; or erythromycin plus gastric lavage	NG gastric lavage alone	Erythromycin arm: EGD 20–60 min after infusion; gastric lavage arm: EGD within 30 min after last irrigation	Satisfactory visualization using Frossard scoring system; score ≥6/8 defined satisfactory visualization
Pateron et al., 2011 (35)	France; 6 emergency departments	Prospective randomized multicenter 3-arm trial	253 randomized/analyzed: erythromycin 84, gastric lavage 85, erythromycin plus gastric lavage 84	IV erythromycin 250 mg over 20 min; or erythromycin plus NG gastric lavage	NG gastric lavage alone	Erythromycin arm: EGD 30 min after infusion; gastric lavage arm: EGD 15 min after last lavage; combined arm received lavage followed by erythromycin as in erythromycin arm	Satisfactory stomach visualization using Frossard scoring system; score ≥6/8 defined satisfactory visualization
Vimonsuntirungsri et al., 2024 (12)	Thailand; 2 centers	Double-blind randomized placebo-controlled trial	68 randomized; 62 analyzed: metoclopramide 31, placebo 31	IV metoclopramide 10 mg in 10 mL normal saline over 5 min	IV placebo normal saline over 5 min	EGD 30–120 min after drug/placebo administration	Adequate visualization using endoscopic visualized gastroduodenal score; EVS ≥6/8 defined adequate visualization

Data are presented as reported in the original randomized controlled trials. For three-arm trials, intervention arms were displayed separately when applicable. Timing before endoscopy refers to the interval between completion or administration of the assigned intervention and performance of upper gastrointestinal endoscopy. In trials using gastric lavage, lavage was considered either a comparator or co-intervention according to the original study design. The primary outcome/assessment column reflects the main endoscopic visualization or gastric-cleansing measure used by each trial.

EGD, esophagogastroduodenoscopy; ERY, erythromycin; GL, gastric lavage; ICU, intensive care unit; IV, intravenous; MET, metoclopramide; NG, nasogastric; UGIB, upper gastrointestinal bleeding; UGIH, upper gastrointestinal hemorrhage.

**Table 2:** Baseline population and clinical characteristics of included participants.

Study	Sample analyzed	Age	Sex	Presentation/population	Baseline severity/clinical profile	Bleeding etiology/endoscopic source
Ali Shah et al., 2020 (28)	60	Overall 53.68 ± 16.64 years; erythromycin 53.13 ± 17.70 vs placebo 54.23 ± 15.80 years	Overall male 36/60 (60%)	Acute UGIB within 12 h; variceal and non-variceal UGIB included	Baseline hemodynamic/laboratory severity details not fully reported in an extractable form	Variceal bleeding 12 vs 14; non-variceal bleeding 18 vs 16; bleeding site: esophagus 11 vs 11, stomach 17 vs 15, duodenum 2 vs 4
Altraif et al., 2011 (29)	90	Erythromycin 62.3 ± 9.8 vs placebo 62.7 ± 14.7 years	Male: 32/47 (68.1%) vs 31/43 (72.1%)	Adult patients with liver cirrhosis/chronic liver disease presenting with hematemesis within 12 h; final analysis restricted to variceal bleeding	Systolic BP 107.7 vs 101.0 mmHg; Child-Pugh-Turcotte score 7.9 vs 8.1; hemoglobin 95.7 vs 92.0 g/L; admission-to-endoscopy: 3.6 vs 3.3 h	Esophageal varices 43 vs 38; fundal varices 3 vs 3; portal gastropathy 1 vs 2
Carbonell et al., 2006 (31)	99	Erythromycin 59.3 ± 14.6 vs placebo 57.0 ± 13.4 years	M/F: 40/9 vs 38/12	UGIB with blood in the stomach confirmed by gastric lavage; hemodynamically stable after resuscitation	Hemoglobin 7.9 ± 2.0 vs 7.9 ± 2.0 g/dL; admission-to-endoscopy 5.48 ± 3.42 vs 4.54 ± 2.58 h; SAPS II 25.9 ± 9.0 vs 26.1 ± 10.2; cirrhosis 35 vs 32	Esophageal varices 30 vs 27; ulcer 13 vs 16; gastritis 1 vs 0; Mallory–Weiss syndrome 2 vs 0; not determined 3 vs 7
Coffin et al., 2002 (32)	41	Erythromycin 56 ± 19 vs control 58 ± 20 years	M/F: 11/8 vs 14/8	Acute UGIB requiring ICU admission and EGD within 12 h	Hemoglobin 7.4 vs 8.0 g/dL; known/presumed cirrhosis 7 vs 12	Endoscopic lesions included duodenal ulcer, esophageal varices, Mallory–Weiss tear, esophagitis, and erosive gastritis
Javad Ehsani Ardakani et al., 2013 (30)	40	Erythromycin 61 ± 15 vs placebo 62 ± 17 years	Male: 11/20 vs 11/20	Acute UGIB admitted within 12 h; hematemesis or melena	Baseline hemoglobin 9.4 ± 1.7 vs 8.3 ± 2.1 g/dL; pre-endoscopy hemoglobin 8.8 ± 1.7 vs 7.7 ± 1.4 g/dL; cirrhosis 2 vs 1	Hematemesis 19/20 vs 19/20; melena 11/20 vs 9/20
Frossard et al., 2002 (33)	105	Erythromycin 59.2 ± 15 vs placebo 64.5 ± 16 years	M/F: 39/12 vs 45/9	Hematemesis within 12 h; patients with NG tube or gastric lavage before endoscopy were excluded	Hemoglobin 8.8 ± 2.4 vs 8.0 ± 1.9 g/dL; bleeding-to-endoscopy delay 6.4 ± 3.7 vs 7.6 ± 5.1 h; cirrhosis 13 vs 19	Gastric ulcer 17 vs 16; duodenal ulcer 15 vs 17; esophageal varix 14 vs 15; peptic esophagitis 2 vs 4; Mallory–Weiss syndrome 2 vs 2; tumor 1 vs 0
Manupeeraphant et al., 2024 (6)	284	Metoclopramide 62.34 ± 13.9 vs placebo 63.31 ± 14.6 years	Male: 98/143 (68.5%) vs 94/141 (66.7%)	Acute UGIH; EGD performed within 24 h after diagnosis	Hemoglobin 8.28 ± 2.7 vs 8.61 ± 2.9 g/dL; cirrhosis 38/143 (26.6%) vs 34/141 (24.1%); Glasgow-Blatchford score 9.24 ± 4.3 vs 8.27 ± 4.2; diagnosis-to-EGD 16.96 ± 6.2 vs 17.93 ± 5.6 h	Variceal bleeding 25 vs 26; non-variceal bleeding 118 vs 115; peptic ulcer 96 vs 89
Na et al., 2017 (34)	43	Erythromycin 60 ± 14, gastric lavage 63 ± 12, erythromycin plus gastric lavage 57 ± 15 years	M/F: 12/2, 13/2, 13/1	Acute non-variceal UGIB; hematemesis or melena within 12 h	Hemoglobin 11.0 ± 3.0, 9.5 ± 3.2, 11.6 ± 3.4 g/dL; Glasgow-Blatchford score 7, 9, 6; pre-endoscopic Rockall score 1, 2, 1	Hematemesis 5, 5, 5; melena 9, 10, 9
Pateron et al., 2011 (35)	253	Erythromycin 61 ± 14, gastric lavage 61 ± 15, erythromycin plus gastric lavage 60 ± 17 years	M/F: 58/26, 62/23, 61/23	Hematemesis or melena within 12 h in emergency department patients	Hemoglobin 8.9, 8.5, 8.4 g/dL; cirrhosis 28/84 (33%), 29/85 (34%), 27/84 (33%); NSAID use 32/84 (38%), 26/85 (31%), 22/84 (27%)	Mixed UGIB population; endoscopic findings included varices, ulcers, gastritis, Mallory–Weiss syndrome, and other causes
Vimonsuntirungsri et al., 2024 (12)	62 analyzed; 68 randomized	Metoclopramide 63.1 ± 15.7 vs placebo 60.2 ± 16.5 years	M/F among randomized participants: 21/13 vs 16/18	Active UGIB defined by hematemesis or fresh blood through NG tube	Cirrhosis 41.2% vs 41.2%; Glasgow-Blatchford score 11.6 ± 3.3 vs 10.0 ± 4.5; hemoglobin 7.5 ± 2.6 vs 9.2 ± 3.4 g/dL; bleeding-to-EGD 10.5 ± 3.2 vs 12.4 ± 4.4 h	Esophageal lesion 13 vs 13; gastric lesion 13 vs 11; duodenal lesion 4 vs 3; indeterminate lesion due to retained clot 1 vs 4

Baseline characteristics are summarized according to the analyzed population in each trial. Continuous variables are presented as mean ± standard deviation unless otherwise stated by the original study. For three-arm studies, values are reported in the order of the intervention arms shown in the table. Bleeding etiology and endoscopic source were extracted as reported by each trial; therefore, categories may differ across studies. Some trials included mixed variceal and non-variceal bleeding populations, whereas others were restricted to variceal or non-variceal upper gastrointestinal bleeding. When data were not fully reported or not extractable, this was indicated as not reported.

ERY, erythromycin; GL, gastric lavage; MET, metoclopramide; M/F, male/female; NG, nasogastric; NR, not reported; NSAID, non-steroidal anti-inflammatory drug; SAPS II, Simplified Acute Physiology Score II; UGIB, upper gastrointestinal bleeding; UGIH, upper gastrointestinal hemorrhage.

### 3.3 Risk of bias assessment

Risk of bias was assessed using the Cochrane Risk of Bias 2 tool for the primary outcome of adequate gastric emptying or adequate endoscopic visualization. Overall, two trials were judged to be at low risk of bias, while eight trials were judged to have some concerns. No included study was judged to be at high risk of bias.

Because adequate gastric emptying or endoscopic visualization was partly based on subjective endoscopist assessment, assessor blinding was re-evaluated for each included trial under RoB 2 Domain 4. Endoscopist or outcome-assessor blinding was clearly reported in Ali Shah et al. (28), Altraif et al. (29), Coffin et al. (32), Frossard et al. (33), Manupeeraphant et al. (6), Na et al. (34), Pateron et al. (35), and Vimonsuntirungsri et al. (12). Carbonell et al. (31) was a double-blind randomized trial with pharmacy-controlled identical treatment allocation, but explicit endoscopist blinding was less directly described; therefore, Domain 4 was retained as low risk because outcome assessment was embedded within a double-blind protocol, but this was noted in the detailed RoB table. Javad Ehsani Ardakani et al. (30) was described as double-blind, but details of assessor blinding and allocation concealment were insufficiently reported; therefore, Domain 4 was judged as some concerns. Overall, the measurement-bias assessment was revised to explicitly document assessor blinding for subjective visualization outcomes.

Frossard et al. (33) and Pateron et al. (35) were judged to have low overall risk of bias. These trials used appropriate randomization procedures, adequate blinding or allocation procedures, and generally complete outcome reporting. Pateron et al. (35) also used central interactive voice-response randomization and an intention-to-treat approach, with missing primary outcome data treated conservatively.

The remaining trials were judged to have some concerns. In several studies, concerns were related to unclear allocation concealment, incomplete reporting of the randomization process, absence of a clearly available prespecified protocol, or post-randomization exclusions. Coffin et al. (32) were endoscopist-blinded but did not use a placebo control, and the ICU team was not blinded. Javad Ehsani Ardakani et al. (30) were described as randomized and double-blind, but details of sequence generation, allocation concealment, and blinding were insufficiently reported. Altraif et al. (29) and Vimonsuntirungsri et al. had some concerns, mainly because some randomized participants were excluded before final analysis. Manupeeraphant et al. (6) was a large double-blind multicenter trial, but the trial registration occurred after enrollment had started, and the final analysis excluded participants with incomplete protocol or refusal to stay after endoscopy.

Overall, the body of evidence was considered methodologically acceptable for quantitative synthesis, but the presence of some concerns in most studies should be considered when interpreting the certainty and robustness of the findings (**Figure 2****) (****Appendix Table A2**).

**Figure 2:**
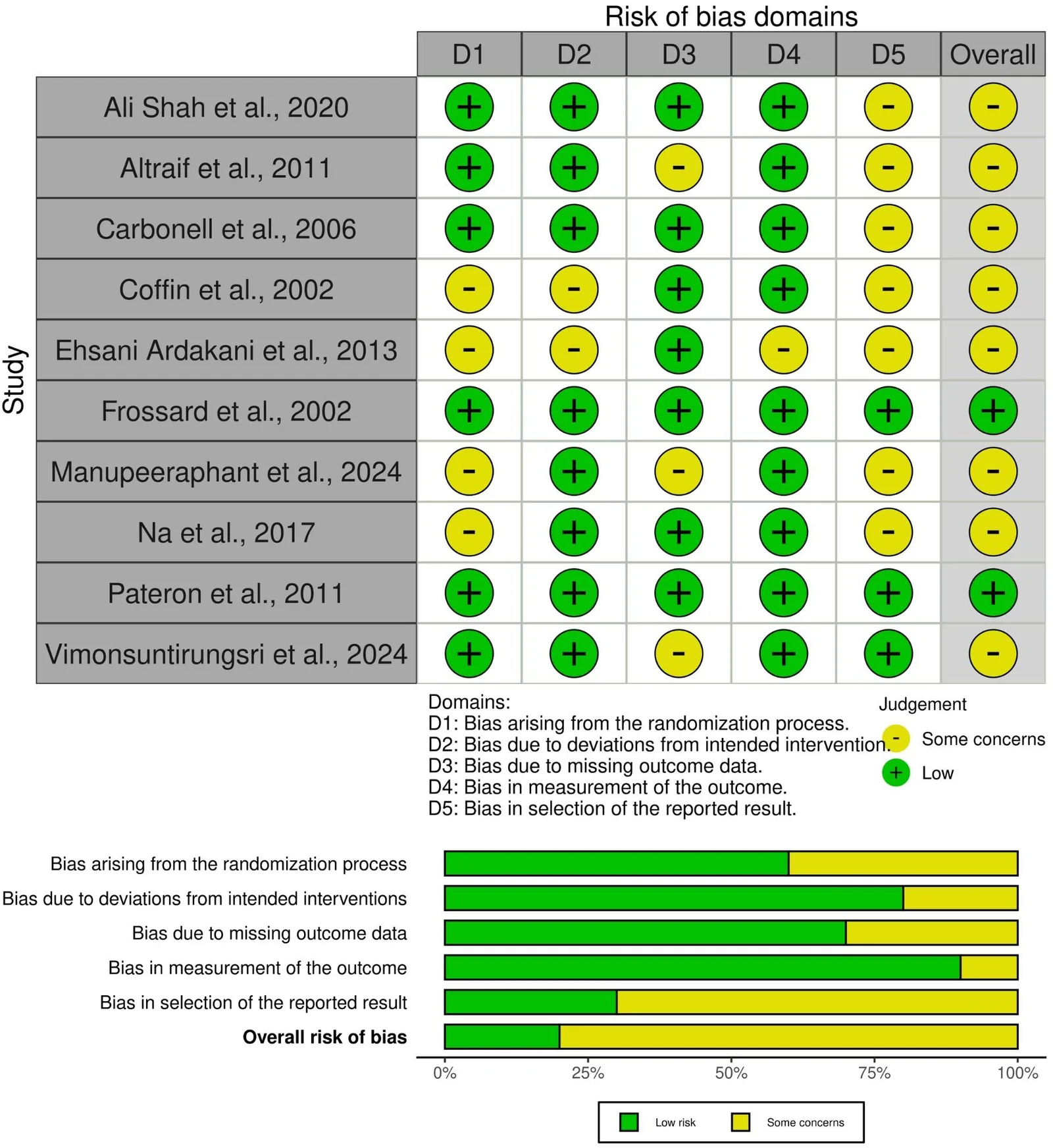
‘Risk of bias graph’: review authors’ judgments about each risk of bias item presented as percentages across all included studies, and ‘Risk of bias summary’: review authors’ judgments about each risk of bias item for each included study.

### 3.4 Outcomes

#### 3.4.1 Adequate gastric emptying/adequate endoscopic visualization

Eleven comparisons, including 793 participants, reported adequate gastric emptying or adequate endoscopic visualization. Prokinetic therapy was associated with a significantly higher likelihood of adequate visualization compared with control. The pooled odds ratio was 3.68 under the random-effects model (95% CI, 2.06–6.60; p < 0.0001), indicating that patients receiving pre-endoscopic prokinetic therapy had more than threefold higher odds of adequate visualization. Moderate heterogeneity was observed across studies (I^2^ = 59.9%; Q = 24.95, p = 0.0054), suggesting variation in treatment effect, likely related to differences in drug type, comparator design, use of gastric lavage, bleeding etiology, and outcome definitions. Leave-one-out sensitivity analysis showed that the effect remained statistically significant after excluding each study individually, supporting the robustness of this finding. (**Figure 3**).

**Figure 3:**
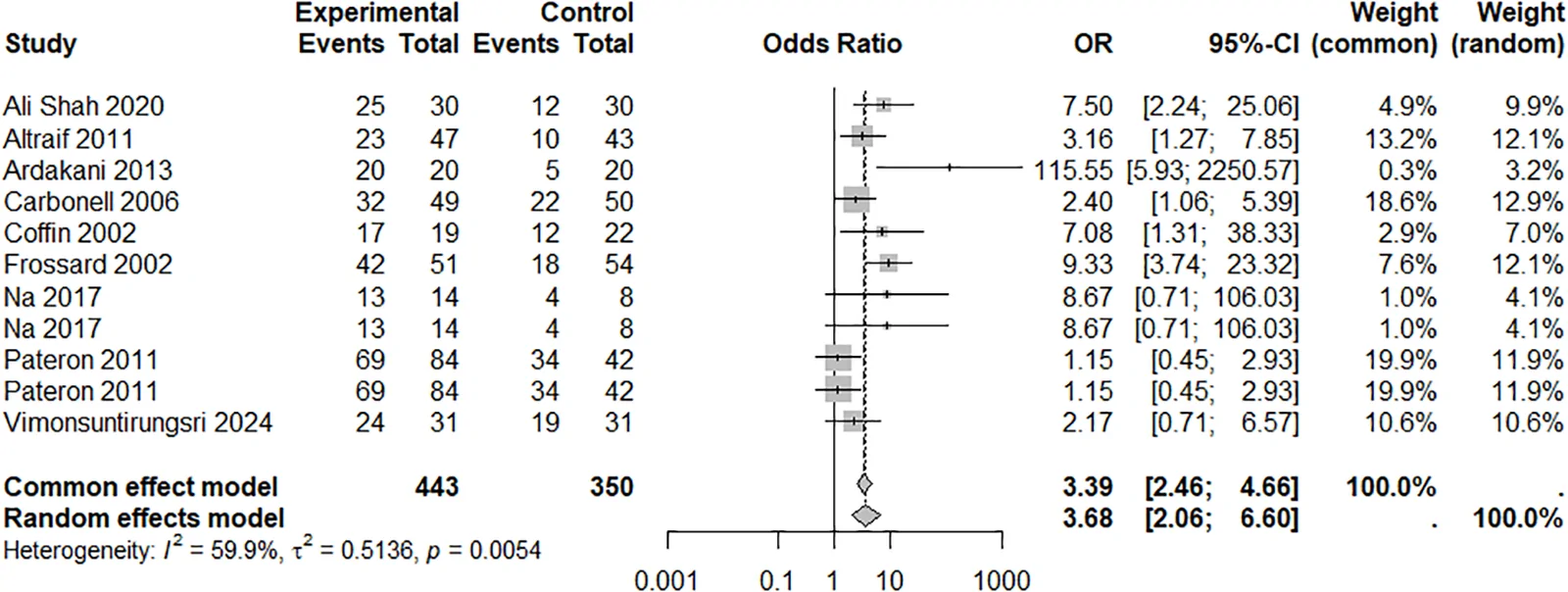
Effect of pre-endoscopic prokinetic therapy on adequate gastric emptying or adequate endoscopic visualization.

#### 3.4.2 Need for second-look/repeat endoscopy

Ten comparisons, including 1,034 participants, reported the need for second-look or repeat endoscopy. Prokinetic therapy significantly reduced the need for repeat endoscopy compared with control. The random-effects pooled odds ratio was 0.54 (95% CI, 0.37–0.79; p = 0.0015), suggesting an approximately 46% reduction in the odds of requiring a second-look or repeat procedure. Heterogeneity was low and not statistically significant (I^2^ = 20.8%; Q = 11.37, p = 0.2513). The leave-one-out analysis demonstrated that the direction and statistical significance of the pooled effect were maintained after removing individual studies, indicating a stable and consistent result (**Figure 4**).

**Figure 4:**
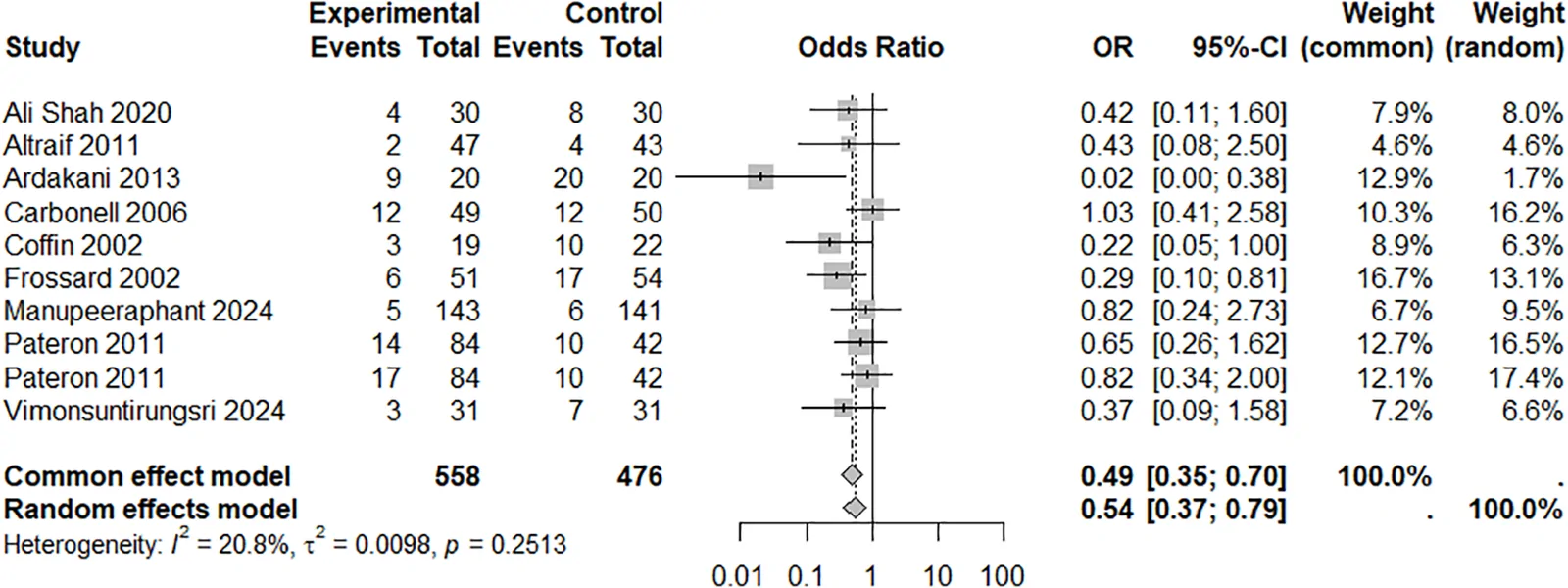
Effect of pre-endoscopic prokinetic therapy on need for second-look or repeat endoscopy.

#### 3.4.3 All-cause mortality

Six studies, including 937 participants, reported all-cause mortality in extractable form. Prokinetic therapy was not associated with a statistically significant reduction in mortality. The random-effects pooled odds ratio was 0.59 (95% CI, 0.32–1.09; p = 0.0904). No heterogeneity was observed (I^2^ = 0%; Q = 1.77, p = 0.8798). Although the point estimate favored prokinetic therapy, the confidence interval crossed the line of no effect, indicating that the available randomized evidence does not demonstrate a clear mortality benefit (**Figure 5**).

**Figure 5:**
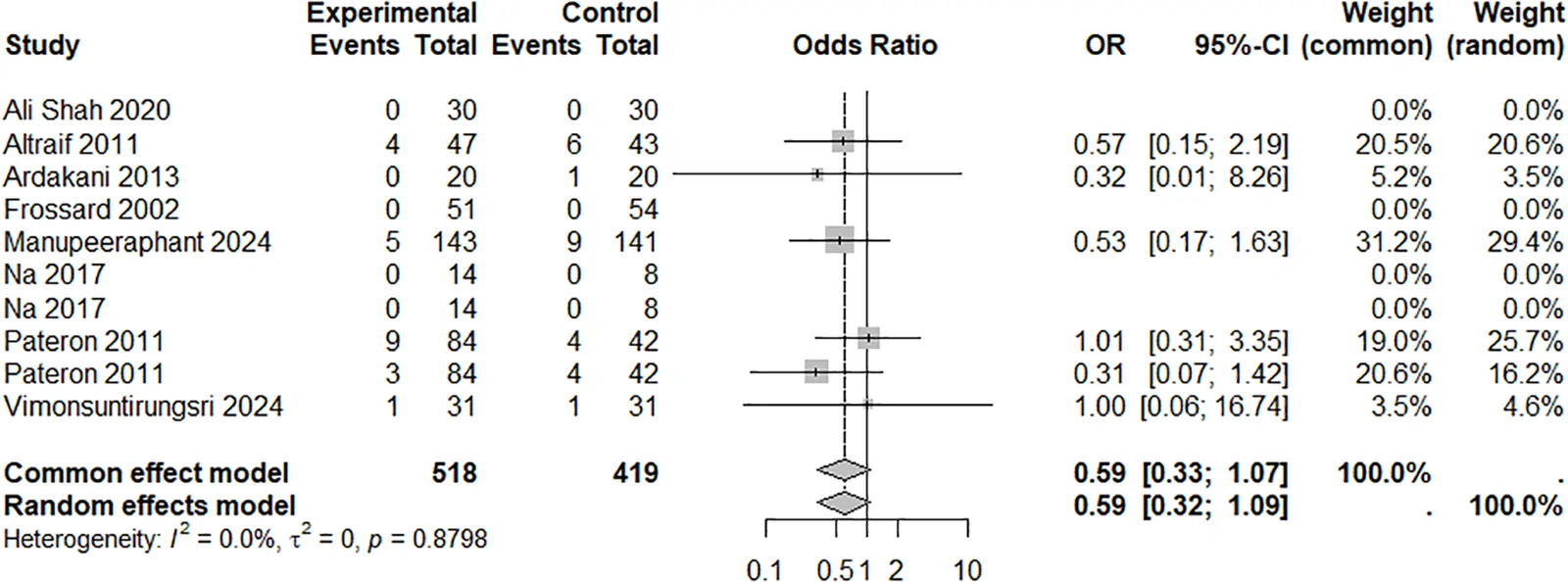
Effect of pre-endoscopic prokinetic therapy on all-cause mortality.

#### 3.4.4 Endoscopy duration

Eleven comparisons, including 1,036 participants, reported endoscopy duration. Under the common-effect model, prokinetic therapy was associated with a statistically significant reduction in procedure duration (MD, −1.45 minutes; 95% CI, −2.37 to −0.53; p = 0.0019). However, the random-effects estimate was not statistically significant (MD, −1.67 minutes; 95% CI, −5.59 to 2.25; p = 0.4046). Heterogeneity was very high (I^2^ = 93.8%; Q = 161.45, p < 0.0001). The leave-one-out analysis suggested that Javad Ehsani Ardakani et al. (30) contributed substantially to heterogeneity, as omission of this study reduced I^2^ to 61.3%. Therefore, although some studies showed shorter procedures with prokinetics, the pooled evidence for endoscopy duration should be interpreted cautiously (**Figure 6**).

**Figure 6:**
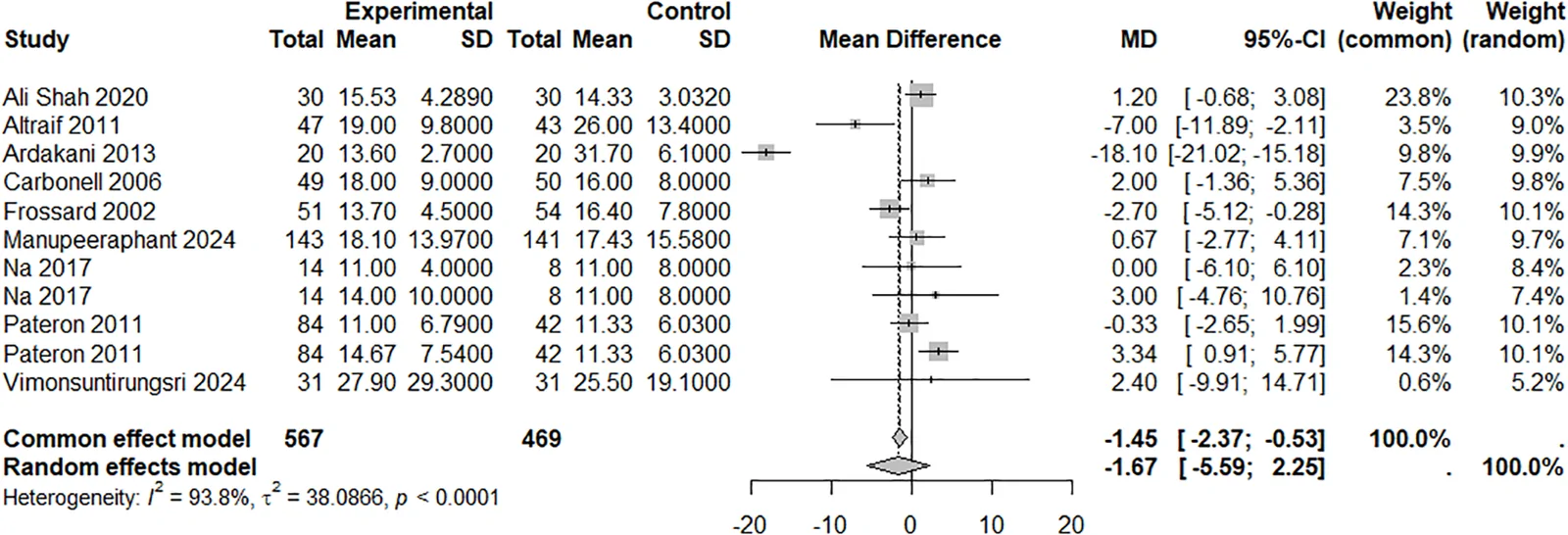
Effect of pre-endoscopic prokinetic therapy on endoscopy duration.

#### 3.4.5 Blood transfusion requirement

Ten comparisons, including 752 participants, reported blood transfusion requirements. The common-effect model suggested a significant reduction in transfused blood units with prokinetic therapy (MD, −0.59 units; 95% CI, −0.88 to −0.31; p < 0.0001). However, the random-effects model did not show a statistically significant difference (MD, −0.64 units; 95% CI, −1.44 to 0.15; p = 0.1126). Substantial heterogeneity was present (I^2^ = 83.5%; Q = 54.60, p < 0.0001). Sensitivity analysis showed that omission of Javad Ehsani Ardakani et al. (30) reduced heterogeneity to 0% and attenuated the effect, suggesting that this study strongly influenced the pooled estimate. Therefore, evidence for reduced transfusion requirement is uncertain (**Figure 7**).

**Figure 7:**
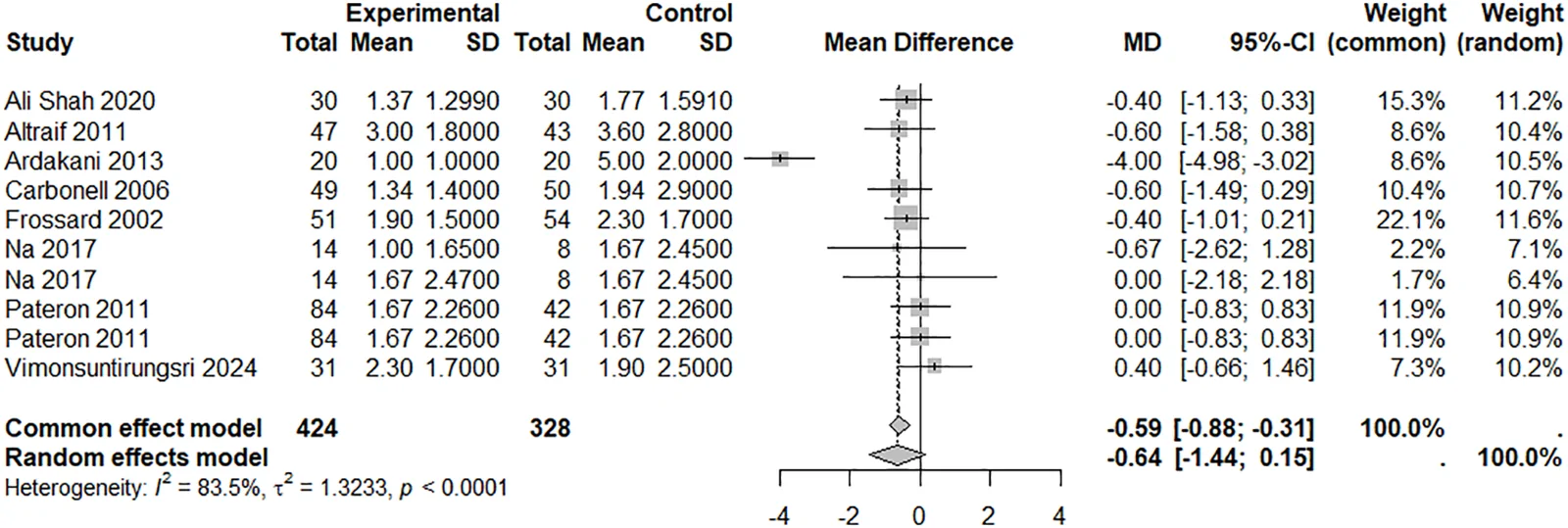
Effect of pre-endoscopic prokinetic therapy on blood transfusion requirement.

#### 3.4.6 Length of hospital stay

Six studies, including 456 participants, reported the length of hospital stay. Prokinetic therapy was associated with a statistically significant reduction in hospital stay. The random-effects pooled mean difference was −1.30 days (95% CI, −2.11 to −0.49; p = 0.0016), suggesting that pre-endoscopic prokinetic therapy shortened hospitalization by approximately 1.3 days. Heterogeneity was low and not statistically significant (I^2^ = 20.8%; Q = 6.32, p = 0.2766). The leave-one-out analysis showed that the result remained statistically significant after excluding each study, supporting the robustness of this outcome (**Figure 8**).

**Figure 8:**
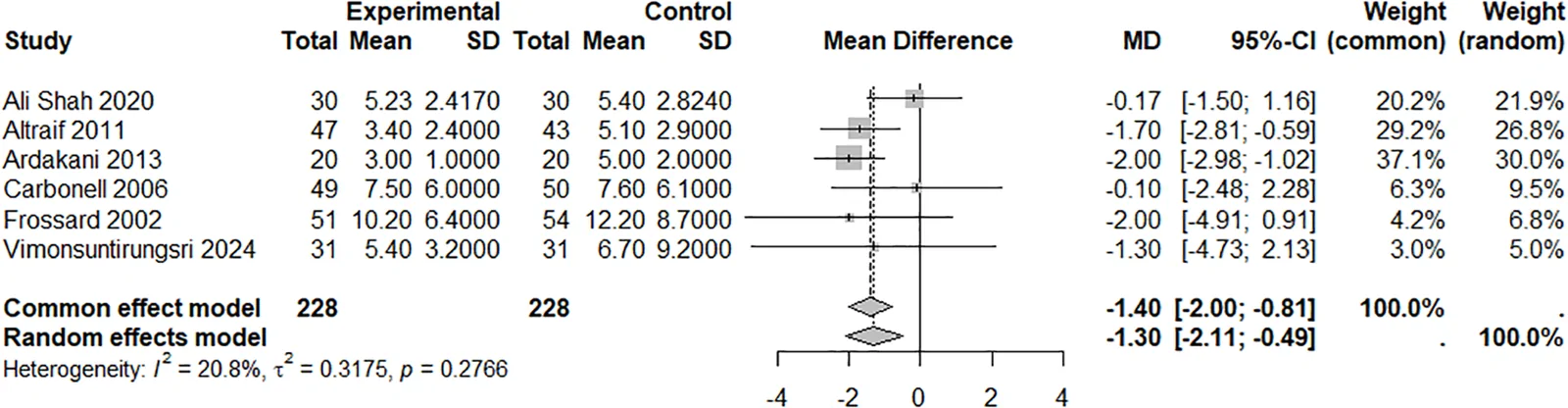
Effect of pre-endoscopic prokinetic therapy on length of hospital stay.

#### 3.4.7 Endoscopic visualization score

Eight comparisons, including 829 participants, reported endoscopic visualization scores. Because the included studies used different scoring systems and scales, including 0–3, 0–8, 0–10, and 0–16 scales, this outcome was reanalyzed using standardized mean difference rather than raw mean difference. Pre-endoscopic prokinetic therapy was associated with significantly improved endoscopic visualization scores under the random-effects model, with a standardized mean difference of 0.47 (95% CI, 0.21–0.72; p = 0.0003). The common-effect estimate was similar in direction and statistically significant (SMD, 0.42; 95% CI, 0.28–0.56; p < 0.0001). Moderate-to-substantial heterogeneity was observed (I^2^ = 62.1%; τ^2^ = 0.0783; Q = 18.45, p = 0.0101). Therefore, although the direction of effect supports improved visualization, this continuous outcome should still be interpreted cautiously because scoring systems differed across trials. The dichotomous adequate-visualization outcome remains the more clinically interpretable endpoint (**Figure 9**).

**Figure 9:**
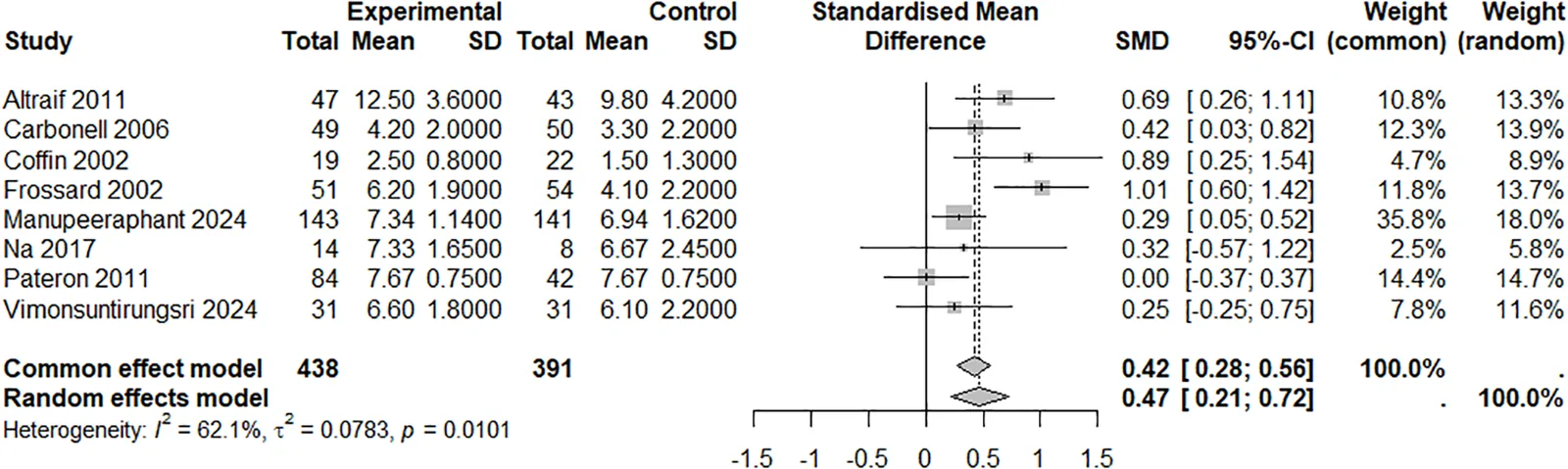
Effect of pre-endoscopic prokinetic therapy on endoscopic visualization score. Because included trials used different visualization scoring systems, the effect was pooled using standardized mean difference with Hedges’ g correction. SMD, standardized mean difference; CI, confidence interval.

#### 3.4.8 Identification of bleeding source/diagnosis established

Six comparisons, including 844 participants, reported successful identification of the bleeding source or establishment of a diagnosis. Prokinetic therapy was associated with a modest but statistically significant improvement in identifying the bleeding source. The random-effects pooled odds ratio was 1.55 (95% CI, 1.00–2.40; p = 0.0499). No heterogeneity was observed (I^2^ = 0%; Q = 3.49, p = 0.6251). However, the effect estimate was borderline, and leave-one-out analysis showed that statistical significance was not consistently maintained after exclusion of some individual studies. Therefore, this finding should be interpreted as suggestive rather than definitive (**Figure 10**).

**Figure 10:**
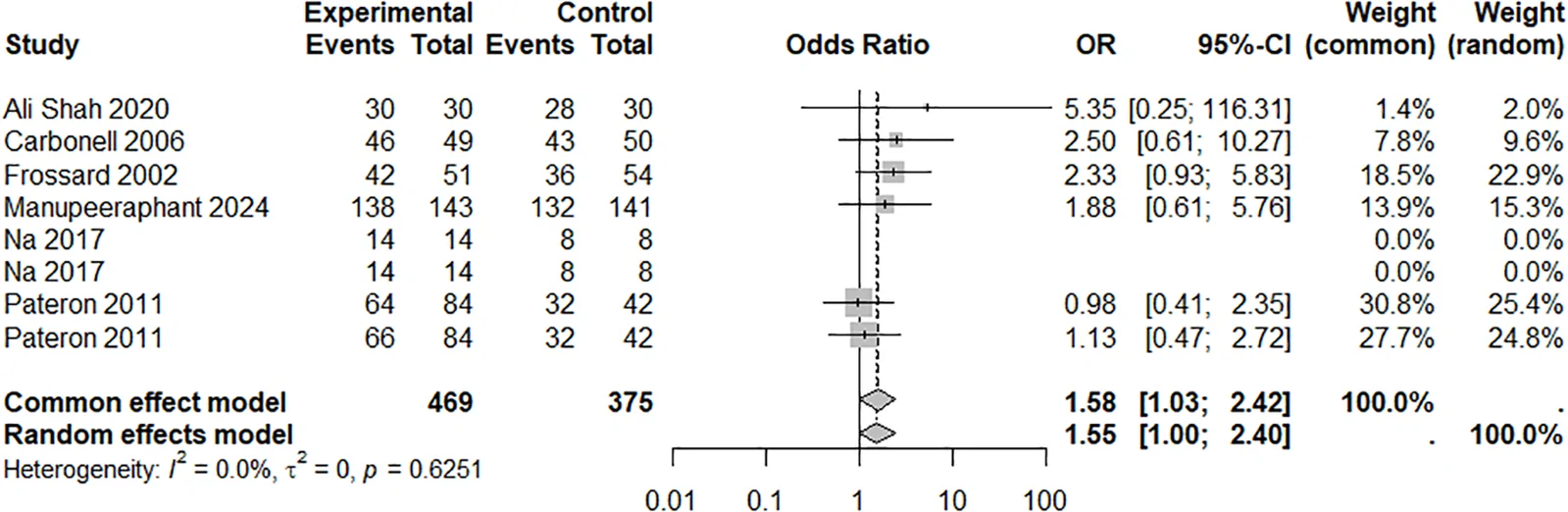
Effect of pre-endoscopic prokinetic therapy on identification of the bleeding source or diagnostic establishment.

#### 3.4.9 Rebleeding

Five comparisons, including 356 participants, reported rebleeding. Prokinetic therapy was not associated with a significant difference in rebleeding risk compared with control. The random-effects pooled odds ratio was 1.12 (95% CI, 0.64–1.97; p = 0.6978). No heterogeneity was observed (I^2^ = 0%; Q = 0.05, p = 0.9997). These findings suggest that although prokinetic therapy improves visualization and reduces repeat endoscopy, current evidence does not show a clear effect on rebleeding (**Figure 11**).

**Figure 11:**
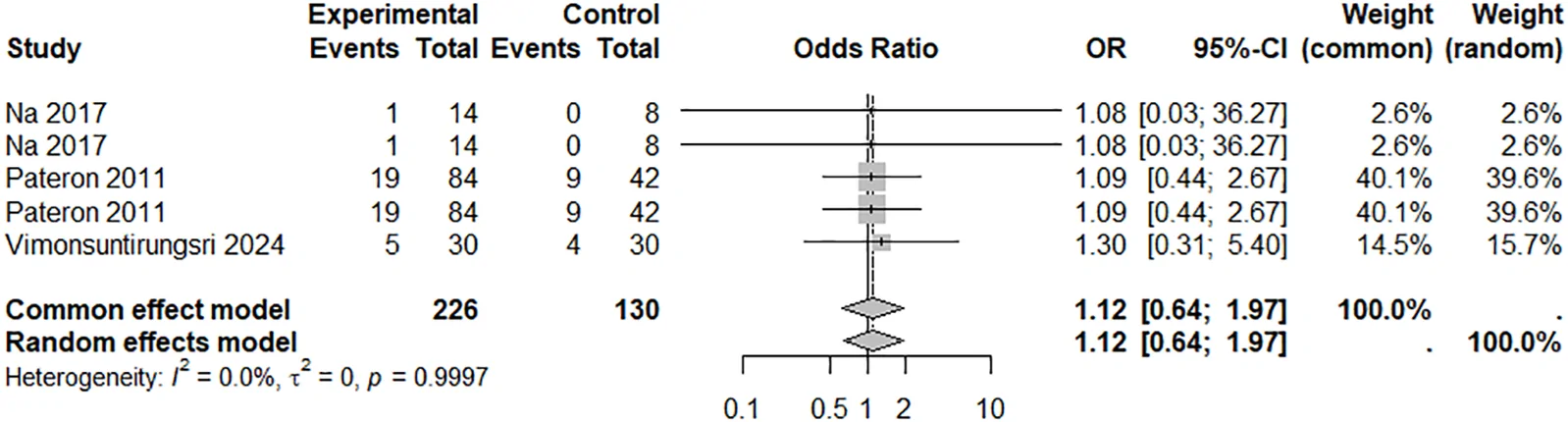
Effect of pre-endoscopic prokinetic therapy on rebleeding.

#### 3.4.10 Safety and adverse events

Safety outcomes were extracted when reported by the included randomized trials. Overall, adverse-event reporting was limited and inconsistent across studies. Several trials reported no drug-related or endoscopy-related adverse events, while others did not provide detailed safety data for specific events of interest. Reported safety outcomes included endoscopy-related complications, bronchopulmonary aspiration, cardiac arrhythmias or QT-related concerns, gastrointestinal symptoms, and drug-related adverse events. Across the included trials, no consistent signal of serious harm was identified; however, the trials were not primarily designed or powered to detect uncommon adverse events such as QT prolongation, clinically significant arrhythmias, or extrapyramidal symptoms. Therefore, the absence of frequent adverse events in the included trials should be interpreted cautiously. A study-level summary of reported adverse events is provided in **Supplementary Table A3**.

##### 3.4.10.1 Summary of findings

Overall, pre-endoscopic prokinetic therapy significantly improved adequate gastric emptying/endoscopic visualization and reduced the need for second-look or repeat endoscopy. It also appeared to reduce length of hospital stay. However, no statistically significant benefit was observed for all-cause mortality or rebleeding. Outcomes such as endoscopy duration and transfusion requirement showed substantial heterogeneity and were not significant under random-effects models, indicating uncertainty. The most consistent and clinically robust benefits were therefore improved visualization and reduced repeat endoscopy.

### 3.5 GRADE assessment

Overall, the certainty of evidence was highest for procedural outcomes directly related to the purpose of pre-endoscopic prokinetic therapy. The evidence suggests that pre-endoscopic prokinetic therapy, driven mainly by erythromycin-based trials, probably improves adequate gastric emptying or endoscopic visualization and probably reduces the need for repeat endoscopy. These findings represent the most reliable and clinically interpretable benefits of treatment.

For continuous visualization scores, the certainty of evidence was rated as low. Although the revised standardized mean difference analysis favored prokinetic therapy, the included trials used different visualization scoring systems, and the pooled SMD is less directly interpretable than the dichotomous adequate-visualization outcome.

The certainty of evidence was very low for hard clinical outcomes, including mortality, rebleeding, blood transfusion requirement, and endoscopy duration. These outcomes were limited by imprecision, substantial heterogeneity, variable definitions, and insufficient data for reliable subgroup analysis or meta-regression. Therefore, no firm conclusions can be drawn regarding the effect of pre-endoscopic prokinetic therapy on these outcomes.

Safety evidence was also rated as very low certainty. Although several included trials reported no adverse events, adverse-event reporting was inconsistent and the trials were not powered to detect uncommon but clinically important harms, such as QT prolongation or arrhythmias with erythromycin, or extrapyramidal symptoms with metoclopramide.

Importantly, the overall evidence base was dominated by erythromycin trials, with only two trials evaluating metoclopramide. Therefore, the findings should not be interpreted as a definitive class effect of all prokinetic drugs. Evidence for metoclopramide remains limited and less consistent, and further adequately powered trials are needed to clarify its efficacy and safety (**Table 3**).

**Table 3:** Summary of findings and GRADE assessment.

Outcome	No. of studies/comparisons	Participants	Effect estimate	Certainty of evidence	Main reasons for certainty judgment	Interpretation
Adequate gastric emptying/adequate endoscopic visualization	10 studies/11 comparisons	793	OR 3.68, 95% CI 2.06 to 6.60; p < 0.0001; I^2^ = 59.9%	Moderate	Downgraded for inconsistency because moderate heterogeneity was present. Publication bias could not be confidently excluded, although this was the only outcome with sufficient comparisons for formal visual assessment.	Pre-endoscopic prokinetic therapy, driven mainly by erythromycin-based evidence, probably improves adequate visualization. This is the most clinically interpretable procedural benefit.
Repeat/second-look endoscopy	9–10 studies/10 comparisons	1,034	OR 0.54, 95% CI 0.37 to 0.79; p = 0.0015; I^2^ = 20.8%	Moderate	Downgraded for indirectness because repeat endoscopy definitions and indications varied across trials.	Prokinetic therapy, mainly erythromycin, probably reduces the need for repeat endoscopy. This benefit is consistent with improved index-endoscopy visualization.
Endoscopic visualization score	8 comparisons	829	SMD 0.47, 95% CI 0.21 to 0.72; p = 0.0003; I^2^ = 62.1%	Low	Downgraded for inconsistency due to moderate-to-substantial heterogeneity and for indirectness because visualization scores were measured using different scales across trials.	Continuous visualization scores favored prokinetic therapy. However, because the outcome was pooled using SMD across different scales, it is less clinically interpretable than the dichotomous adequate-visualization endpoint.
Endoscopy duration	10–11 comparisons	1,036	Random-effects MD −1.67 minutes, 95% CI −5.59 to 2.25; p = 0.4046; I^2^ = 93.8%	Very low	Downgraded for serious inconsistency due to very high heterogeneity, imprecision because the random-effects CI crossed the null, and limited ability to explore heterogeneity because of insufficient studies for reliable subgroup analysis or meta-regression.	Evidence is very uncertain. Current data do not demonstrate a clear reduction in procedure duration.
Blood transfusion requirement	10 comparisons	752	Random-effects MD −0.64 units, 95% CI −1.44 to 0.15; p = 0.1126; I^2^ = 83.5%	Very low	Downgraded for serious inconsistency, imprecision, and indirectness because transfusion thresholds, timing of measurement, bleeding severity, and co-interventions varied across trials.	Evidence is very uncertain. No firm conclusion can be drawn regarding transfusion reduction.
Length of hospital stay	6 comparisons	456	MD −1.30 days, 95% CI −2.11 to −0.49; p = 0.0016; I^2^ = 20.8%	Low	Downgraded for indirectness because hospital discharge practices and care pathways vary by setting, and for imprecision due to limited sample size.	Prokinetic therapy may reduce length of stay, but this finding should be interpreted cautiously because LOS is influenced by healthcare-system factors beyond endoscopic visualization.
Identification of bleeding source	8 comparisons	844	OR 1.55, 95% CI 1.00 to 2.40; p = 0.0499; I^2^ = 0%	Low	Downgraded for imprecision because the confidence interval was borderline and close to no effect, and for indirectness because source identification depends on bleeding etiology, timing of endoscopy, and endoscopist factors.	Prokinetic therapy may improve identification of the bleeding source, but the result is borderline and should be interpreted cautiously.
Rebleeding	5 comparisons	356	OR 1.12, 95% CI 0.64 to 1.97; p = 0.6978; I^2^ = 0%	Very low	Downgraded for serious imprecision because of few events and wide confidence intervals, and for indirectness because rebleeding definitions and follow-up periods varied.	Evidence is very uncertain. Current data do not show a clear effect on rebleeding.
Mortality	8–10 comparisons	937	OR 0.59, 95% CI 0.32 to 1.09; p = 0.0904; I^2^ = 0%	Very low	Downgraded for serious imprecision because events were few and the CI crossed the null, and for indirectness because included trials were not powered to detect mortality differences.	Evidence is very uncertain. Current data do not demonstrate a clear mortality benefit.
Safety and adverse events	10 studies	1,077	Not pooled because adverse-event definitions and reporting were inconsistent	Very low	Downgraded for serious imprecision and reporting limitations. Most trials were designed to assess procedural efficacy rather than rare harms, and safety outcomes were inconsistently defined and reported.	No consistent serious safety signal was identified, but firm conclusions cannot be drawn regarding uncommon events such as QT prolongation, arrhythmia, or extrapyramidal symptoms.

CI, confidence interval; GRADE, Grading of Recommendations Assessment, Development and Evaluation; MD, mean difference; OR, odds ratio.

## 4 Discussion

### 4.1 Principal findings

In this systematic review and meta-analysis of 10 randomized controlled trials including 1,077 adults with acute upper gastrointestinal bleeding, pre-endoscopic prokinetic therapy improved adequate gastric emptying or endoscopic visualization. This finding was driven mainly by erythromycin-based evidence, because eight of the ten included randomized trials evaluated erythromycin. Pre-endoscopic prokinetic therapy also reduced the need for second-look or repeat endoscopy. These findings should be interpreted primarily as procedural benefits related to improved quality of the index endoscopic examination.

The benefit for visualization is clinically relevant because retained blood, clots, and gastric contents may obscure the bleeding source and reduce the diagnostic and therapeutic quality of emergency endoscopy. Improved visualization may facilitate identification of the bleeding lesion, improve diagnostic confidence, and reduce the need for repeat procedures. However, current randomized evidence does not demonstrate a clear benefit for mortality, rebleeding, transfusion requirement, or endoscopy duration. These harder clinical outcomes are influenced by multiple factors beyond gastric clearance, including bleeding etiology, hemodynamic instability, comorbidity burden, quality of resuscitation, endoscopic hemostasis, pharmacologic therapy, and access to rescue interventions (1–4).

Endoscopy duration and blood transfusion requirement showed substantial heterogeneity and were not statistically significant under random-effects models. Potential sources of heterogeneity included differences in prokinetic agent, dose, route of administration, timing before endoscopy, use of gastric lavage, bleeding etiology, transfusion thresholds, baseline severity, and outcome definitions. The available number of studies and comparisons was insufficient to support reliable subgroup analysis or meta-regression for these highly heterogeneous outcomes. Therefore, these outcomes should be interpreted cautiously and considered exploratory rather than confirmatory.

### 4.2 Comparison with previous evidence

Our findings are broadly consistent with earlier meta-analyses showing that erythromycin improves gastric visualization before endoscopy in acute upper gastrointestinal bleeding. Barkun et al. reported that prokinetic therapy improved endoscopic visualization and reduced the need for repeat endoscopy, but the evidence base at that time was limited and largely erythromycin-focused (7). Similarly, Theivanayagam et al. and Rahman et al. found that pre-endoscopic erythromycin improved endoscopic visualization and reduced repeat endoscopy, although both reviews were limited by the small number of available randomized trials and lack of robust evidence for hard clinical outcomes (8, 9).

The 2023 Cochrane review also concluded that erythromycin before endoscopy may improve gastric visualization and may reduce repeat endoscopy, but emphasized limitations related to small study numbers, heterogeneity, and certainty of evidence (5). Our review extends this earlier evidence by incorporating newer randomized trials, including the recent metoclopramide trials, and by evaluating a broader range of outcomes, including mortality, rebleeding, transfusion requirement, length of hospital stay, identification of bleeding source, and adverse events (6, 12).

Recent network meta-analyses have compared erythromycin, metoclopramide, nasogastric lavage, and combined strategies. Pasam et al. reported that erythromycin was more effective than metoclopramide or gastric lavage alone for improving adequate mucosal visualization and was associated with favorable effects on repeat endoscopy and hospitalization duration (10). The 2026 network meta-analysis by Altawil et al. similarly found that erythromycin-based strategies ranked highly for improving endoscopic visualization, whereas evidence for metoclopramide remained limited and less consistent (36). Our findings support these conclusions because the overall beneficial effect was mainly driven by erythromycin trials, while evidence for metoclopramide and for hard clinical outcomes remains uncertain.

### 4.3 Interpretation of the visualization benefit

The improvement in endoscopic visualization is biologically plausible. Erythromycin is a motilin receptor agonist that stimulates gastric antral contractions and accelerates gastric emptying, thereby helping clear retained blood, clots, and gastric contents before endoscopy (5, 7). This mechanism directly addresses one of the main technical barriers to effective endoscopy in acute upper gastrointestinal bleeding: poor visualization of the gastric and duodenal mucosa.

Improved gastric clearance may explain the reduction in repeat endoscopy observed in this review. A clearer stomach may improve diagnostic confidence, facilitate identification of the bleeding source, and support timely endoscopic therapy. However, improved visualization does not necessarily translate into lower mortality or rebleeding. Mortality and rebleeding are influenced by bleeding etiology, hemodynamic status, comorbidities, resuscitation quality, endoscopic hemostasis, pharmacologic therapy, and access to rescue interventions (1–4).

The continuous visualization-score outcome also favored treatment after reanalysis using standardized mean difference. This approach was methodologically more appropriate than raw mean difference because included studies used different visualization scoring scales. However, the standardized mean difference expresses the effect in standard deviation units and is less clinically interpretable than a direct point difference on a single scale. Therefore, the dichotomous adequate-visualization outcome remains the more clinically meaningful endpoint.

### 4.4 Erythromycin, metoclopramide, and gastric lavage

The findings of this review should not be interpreted as a uniform class effect of all prokinetic drugs. Most included trials evaluated erythromycin, whereas only two trials evaluated metoclopramide. Therefore, the pooled estimates are largely driven by erythromycin-based evidence. The role of metoclopramide remains uncertain because the available trials are few, differ in patient populations, and show less consistent results.

Manupeeraphant et al. reported improvement in endoscopic mucosal visualization scores with intravenous metoclopramide, particularly in selected subgroups (6). In contrast, Vimonsuntirungsri et al. did not show a statistically significant improvement in overall adequate visualization, although the trial suggested possible benefit in gastric-lesion subgroups and reduced second-look endoscopy (12). These findings suggest that metoclopramide may have selective utility in settings where erythromycin is unavailable or contraindicated. However, current evidence is insufficient to support metoclopramide as an evidence-equivalent substitute for erythromycin in all patients with acute upper gastrointestinal bleeding.

The role of gastric lavage also remains debated. Gastric lavage may mechanically remove retained blood and clots, but it is uncomfortable, time-consuming, and may be associated with tube-related adverse effects or aspiration risk. The three-arm trials by Na et al. and Pateron et al. allowed comparison of erythromycin-based strategies against gastric lavage alone (34, 35). Overall, erythromycin-based strategies may provide adequate visualization without routine nasogastric lavage in selected patients. Combined erythromycin and lavage may still be considered in selected patients with severe clot burden or ongoing active bleeding, but current evidence does not support routine lavage for all patients.

### 4.5 Clinical implications

The current evidence most strongly supports selective use of pre-endoscopic intravenous erythromycin in patients with suspected retained blood, clot burden, hematemesis, ongoing active bleeding, or anticipated poor visualization. In these settings, improving gastric clearance before endoscopy may improve the diagnostic and therapeutic quality of the index procedure and reduce the need for repeat endoscopy.

Routine administration of prokinetic therapy to all patients with acute upper gastrointestinal bleeding is not supported by the available evidence. Patients with low-risk presentations, no hematemesis, no evidence of retained gastric blood, or delayed presentation after bleeding has stopped may derive less benefit. Current guideline recommendations support erythromycin in selected patients with clinically severe or ongoing active upper gastrointestinal bleeding rather than universal administration (3, 4). Our findings support this selective approach.

Metoclopramide should not be considered a fully evidence-equivalent substitute for erythromycin based on current data. Its use may be reasonable in settings where erythromycin is unavailable or contraindicated, but this should be interpreted cautiously because metoclopramide evidence remains limited and less consistent. The absence of a clear mortality or rebleeding benefit does not mean that prokinetic therapy lacks clinical value. Rather, the main benefit is improvement in the procedural quality of the index endoscopy.

Implementation should consider timing, contraindications, and local workflow. Intravenous erythromycin requires administration before endoscopy, usually with sufficient time to allow gastric motility effects. Clinicians should also consider baseline QT risk, electrolyte abnormalities, cardiac comorbidities, and concomitant QT-prolonging medications when using erythromycin. For metoclopramide, clinicians should consider risk factors for extrapyramidal reactions or other neurologic adverse effects.

### 4.6 Certainty of evidence

Using the GRADE approach, certainty of evidence was highest for procedural outcomes directly related to the rationale for pre-endoscopic prokinetic therapy (37). Evidence was moderate for adequate gastric emptying or endoscopic visualization and repeat endoscopy. These outcomes suggest that pre-endoscopic prokinetic therapy, mainly erythromycin, probably improves visualization and probably reduces repeat endoscopy.

Certainty was low for continuous visualization score because different trials used different scoring systems. Although the standardized mean difference analysis favored treatment, the result is less clinically interpretable than the dichotomous adequate-visualization outcome. Certainty was also low for length of hospital stay because this outcome may be influenced by healthcare-system factors, admission practices, discharge criteria, and bleeding severity.

Certainty was very low for mortality, rebleeding, endoscopy duration, transfusion requirement, and safety outcomes. These outcomes were limited by imprecision, substantial heterogeneity, sparse events, inconsistent definitions, and insufficient data for reliable subgroup analysis or meta-regression. Safety evidence was limited because adverse-event definitions and monitoring methods were inconsistent across trials. The included trials were not powered to detect rare harms such as QT prolongation, arrhythmia, or extrapyramidal reactions.

### 4.7 Strengths

This review has several strengths. First, it used a comprehensive search across five major databases and included only randomized controlled trials, reducing the risk of confounding compared with observational evidence. Second, it incorporated both older erythromycin trials and newer metoclopramide trials, providing an updated synthesis of currently available pharmacologic options. Third, it evaluated a broad range of outcomes, including adequate visualization, repeat endoscopy, mortality, endoscopy duration, transfusion requirement, length of hospital stay, identification of bleeding source, rebleeding, and adverse events.

Fourth, multi-arm trials were handled by splitting shared comparator groups to avoid double-counting participants in pairwise meta-analyses. Fifth, visualization scores were reanalyzed using standardized mean difference because included studies used different scoring systems. Sixth, risk of bias was reassessed with particular attention to blinding of endoscopists or outcome assessors for subjective visualization outcomes. Finally, certainty of evidence was evaluated using GRADE, allowing cautious interpretation of the strength and limitations of the evidence (37).

### 4.8 Limitations

This review has several limitations. First, although only randomized controlled trials were included, the number of eligible studies was modest, and several outcomes were informed by a small number of trials or comparisons. This limited the precision of several pooled estimates and reduced the ability to conduct robust subgroup analyses or meta-regression.

Second, the evidence base was dominated by erythromycin trials. Eight of the ten included trials evaluated erythromycin, whereas only two evaluated metoclopramide. Therefore, the overall pooled estimates are largely driven by erythromycin-based evidence and should not be interpreted as definitive evidence of a uniform prokinetic class effect. The limited number of metoclopramide trials prevented reliable agent-specific pooled analyses, and conclusions regarding metoclopramide remain uncertain.

Third, substantial heterogeneity was present for some continuous outcomes, particularly endoscopy duration and blood transfusion requirement. Potential sources of heterogeneity include differences in drug type, route of administration, gastric lavage use, bleeding etiology, transfusion thresholds, timing of outcome measurement, baseline severity of bleeding, and co-interventions. However, the available number of studies and comparisons was insufficient to support reliable subgroup analysis or meta-regression. Therefore, these pooled estimates should be considered exploratory, and no firm conclusions should be drawn regarding the effect of pre-endoscopic prokinetic therapy on procedure duration or transfusion requirement.

Fourth, some continuous outcomes required methodological assumptions. Visualization scores were measured using different scoring systems across trials; therefore, this outcome was reanalyzed using standardized mean difference rather than raw mean difference. Although this approach is more appropriate for different scales, it expresses the effect in standard deviation units and is less directly clinically interpretable than the dichotomous adequate-visualization outcome. In addition, some continuous data required conversion from medians and interquartile ranges or ranges to means and standard deviations using established methods. These conversions assume approximate symmetry of the underlying data and may be less reliable when distributions are skewed.

Fifth, safety evidence was limited and inconsistently reported. Most included trials were designed to evaluate procedural efficacy outcomes, such as visualization quality or repeat endoscopy, rather than rare adverse events. Although several studies reported no adverse events, adverse-event definitions, monitoring methods, and reporting completeness varied across trials. Therefore, the current evidence does not allow firm conclusions regarding uncommon but clinically important harms, such as QT prolongation or arrhythmias with erythromycin, or extrapyramidal reactions with metoclopramide.

Sixth, potential publication bias and small-study effects should be considered. Formal assessment of funnel plot asymmetry was feasible only for the primary outcome of adequate gastric emptying or adequate endoscopic visualization because most secondary outcomes included too few studies or comparisons for reliable assessment. Therefore, publication bias could not be confidently excluded, particularly for outcomes with limited evidence, imprecision, or heterogeneous reporting.

Finally, the included trials differed in clinical setting, timing of endoscopy, bleeding etiology, use of nasogastric lavage, route and timing of prokinetic administration, and definitions of outcomes such as repeat endoscopy and length of hospital stay. These differences may limit generalizability across emergency departments, intensive care units, and endoscopy services with different resources and treatment pathways. Accordingly, the findings should be interpreted as strongest for selected patients in whom improved gastric clearance before endoscopy is clinically important, rather than as evidence supporting universal use of pre-endoscopic prokinetic therapy in all patients with acute upper gastrointestinal bleeding.

### 4.9 Recommendations for future research

Future randomized trials should focus on clinically relevant subgroups most likely to benefit from prokinetic therapy, including patients with hematemesis, active bleeding, retained gastric clot, variceal bleeding, or high-risk non-variceal bleeding. Direct head-to-head trials comparing erythromycin and metoclopramide are needed, especially in settings where erythromycin is unavailable or contraindicated. Future studies should standardize visualization scoring systems, definitions of repeat endoscopy, transfusion thresholds, adverse event reporting, and follow-up periods for mortality and rebleeding.

Further research should also evaluate whether combining prokinetic therapy with selective gastric lavage improves outcomes compared with prokinetic therapy alone in patients with severe clot burden. Trials should systematically report safety outcomes, including QT prolongation, arrhythmia, aspiration, extrapyramidal symptoms, and drug interactions. Cost-effectiveness analyses would also be valuable to determine whether selective use of pre-endoscopic prokinetics reduces healthcare resource use by decreasing repeat endoscopy and shortening hospital stay.

## 5 Conclusion

Pre-endoscopic prokinetic therapy improves adequate gastric emptying and endoscopic visualization in adults with acute upper gastrointestinal bleeding and reduces the need for second-look or repeat endoscopy. The evidence also suggests a reduction in length of hospital stay. However, current randomized evidence does not demonstrate a clear benefit for all-cause mortality, rebleeding, blood transfusion requirement, or endoscopy duration under random-effects models. The most consistent clinical value of prokinetic therapy is improvement of the index endoscopic examination. Intravenous erythromycin remains the best-supported agent, whereas the role of metoclopramide requires further confirmation in larger, well-designed randomized trials.

## Data Availability

The original contributions presented in the study are included in the article/**Supplementary Material**, further inquiries can be directed to the corresponding author/s.

## References

[1] Saydam ŞS Molnar M Vora P . The global epidemiology of upper and lower gastrointestinal bleeding in general population: a systematic review. World J Gastrointest Surg. (2023) 15:723–39. doi: 10.4240/wjgs.v15.i4.723 37206079 PMC10190726

[2] Sverdén E Markar SR Agreus L Lagergren J . Acute upper gastrointestinal bleeding. BMJ. (2018) 363:k4023. 10.1136/bmj.k4023 30361444

[3] Laine L Barkun AN Saltzman JR Martel M Leontiadis GI . ACG clinical guideline: upper gastrointestinal and ulcer bleeding. Am J Gastroenterol. (2021) 116:899–917. doi: 10.14309/ajg.0000000000001245 33929377

[4] Gralnek IM Stanley AJ Morris AJ Camus M Lau J Lanas A . Endoscopic diagnosis and management of nonvariceal upper gastrointestinal hemorrhage (NVUGIH): European Society of Gastrointestinal Endoscopy (ESGE) Guideline - Update 2021. Endoscopy. (2021) 53:300–32. doi: 10.1055/a-1369-5274 33567467

[5] Adão D Gois AF Pacheco RL Pimentel CF Riera R . Erythromycin prior to endoscopy for acute upper gastrointestinal haemorrhage. Cochrane Database Syst Rev. (2023) 2:CD013176. 10.1002/14651858.CD013176.pub2 PMC9891197 36723439

[6] Manupeeraphant P Wanichagool D Songlin T Thanathanee P Chalermsuksant N Techathuvanan K . Intravenous metoclopramide for increasing endoscopic mucosal visualization in patients with acute upper gastrointestinal bleeding: a multicenter, randomized, double-blind, controlled trial. Sci Rep. (2024) 14:7598. doi: 10.1038/s41598-024-57913-2 38556533 PMC10982284

[7] Barkun AN Bardou M Martel M Gralnek IM Sung JJY . Prokinetics in acute upper GI bleeding: a meta-analysis. Gastrointest Endosc. (2010) 72:1138–45. doi: 10.1016/j.gie.2010.08.011 20970794

[8] Theivanayagam S Lim RG Cobell WJ Gowda JT Matteson ML Choudhary A . Administration of erythromycin before endoscopy in upper gastrointestinal bleeding: a meta-analysis of randomized controlled trials. Saudi J Gastroenterol. (2013) 19:205–10. doi: 10.4103/1319-3767.118120 24045593 PMC3793471

[9] Rahman R Nguyen DL Sohail U Almashhrawi AA Ashraf I Puli SR . Pre-endoscopic erythromycin administration in upper gastrointestinal bleeding: an updated meta-analysis and systematic review. Ann Gastroenterol. (2016) 29:312–7. doi: 10.20524/aog.2016.0045 27366031 PMC4923816

[10] Pasam RT Bains K Chava S Mohan BP . Pre-endoscopy erythromycin versus metoclopramide for upper gastrointestinal bleeding: a systematic review and network meta-analysis. Clin Endosc. (2025) 58:831–42. doi: 10.5946/ce.2024.351 40611560 PMC12933551

[11] Aziz M Haghbin H Gangwani MK Weissman S Patel AR Randhawa MK . Erythromycin improves the quality of esophagogastroduodenoscopy in upper gastrointestinal bleeding: a network meta-analysis. Dig Dis Sci. (2023) 68:1435–46. doi: 10.1007/s10620-022-07698-z 36112271

[12] Vimonsuntirungsri T Thungsuk R Nopjaroonsri P Faknak N Pittayanon R . The efficacy of metoclopramide for gastric visualization by endoscopy in patients with active upper gastrointestinal bleeding: double-blind randomized controlled trial. Am J Gastroenterol. (2024) 119:846–55. doi: 10.14309/ajg.0000000000002620 38059896

[13] Cochrane Handbook for Systematic Reviews of Interventions Cochrane Training (2024). Available online at: https://training.cochrane.org/handbook/current (Accessed August 17, 2024).

[14] Page MJ McKenzie JE Bossuyt PM Boutron I Hoffmann TC Mulrow CD . The PRISMA 2020 statement: an updated guideline for reporting systematic reviews. BMJ. (2021) 372:n71. doi: 10.31222/osf.io/v7gm2 33782057 PMC8005924

[15] Ouzzani M Hammady H Fedorowicz Z Elmagarmid A . Rayyan-a web and mobile app for systematic reviews. Syst Rev. (2016) 5:210. doi: 10.1186/s13643-016-0384-4 27919275 PMC5139140

[16] Sterne JAC Savović J Page MJ Elbers RG Blencowe NS Boutron I . RoB 2: a revised tool for assessing risk of bias in randomised trials. BMJ. (2019) 366:l4898. doi: 10.1136/bmj.l4898 31462531

[17] McGuinness LA Higgins JPT . Risk-of-bias VISualization (robvis): an R package and Shiny web app for visualizing risk-of-bias assessments. Res Synth Methods. (2021) 12:55–61. doi: 10.1002/jrsm.1411 32336025

[18] Egger M Smith G Schneider M Minder C . Bias in meta-analysis detected by a simple, graphical test. BMJ. (1997) 315:629–34. doi: 10.1136/bmj.315.7109.629 9310563 PMC2127453

[19] Kanters S . Fixed- and random-effects models. Methods Mol Biol. (2022) 2345:41–65. doi: 10.1007/978-1-0716-1566-9_3 34550583

[20] DerSimonian R Laird N . Meta-analysis in clinical trials. Control Clin Trials. (1986) 7:177–88. doi: 10.1016/j.cct.2015.09.002 3802833

[21] Mantel N Haenszel W . Statistical aspects of the analysis of data from retrospective studies of disease. J Natl Cancer Inst. (1959) 22:719–48. 13655060

[22] Higgins JPT Thompson SG . Quantifying heterogeneity in a meta-analysis. Stat Med. (2002) 21:1539–58. doi: 10.1191/026921699666884611 12111919

[23] Viechtbauer W . Bias and efficiency of meta-analytic variance estimators in the random-effects model. J Educ Behav Stat. (2005) 30:261–93. doi: 10.3102/1076998603000326138293548

[24] Altman DG Bland JM . Standard deviations and standard errors. BMJ. (2005) 331:903. doi: 10.1136/bmj.331.7521.903 16223828 PMC1255808

[25] Higgins JPT Thomas J Chandler J Cumpston M Li T Page MJ . Cochrane Handbook for Systematic Reviews of Interventions Version 6.3 (Updated February 2022) (2022). Cochrane. Available online at: https://training.cochrane.org/handbook (Accessed May 7, 2023).

[26] Rosenthal R Rubin DB . Meta-analytic procedures for combining studies with multiple effect sizes. Psychol Bull. (1986) 99:400–6. doi: 10.1037/0033-2909.99.3.40027371692

[27] Bergh D . Sample size and chi-squared test of fit—a comparison between a random sample approach and a chi-square value adjustment method using Swedish adolescent data. In: Zhang Q Yang H , editors. Pacific Rim Objective Measurement Symposium (Proms) 2014 Conference Proceedings. Springer Berlin Heidelberg, Berlin, Heidelberg (2015). p. 197–211.

[28] Ali Shah SA Nadeem M Jameel M Yasmin R Afsar A Riaz F . Oral erythromycin improves the quality of endoscopy in upper gastrointestinal bleeding patients. Cureus. (2020) 12:e10204. doi: 10.7759/cureus.10204 33042657 PMC7534507

[29] Altraif I Handoo FA Aljumah A Alalwan A Dafalla M Saeed AM . Effect of erythromycin before endoscopy in patients presenting with variceal bleeding: a prospective, randomized, double-blind, placebo-controlled trial. Gastrointest Endosc. (2011) 73:245–50. doi: 10.1016/j.gie.2010.09.043 21145052

[30] Javad Ehsani Ardakani M Zare E Basiri M Mohaghegh Shalmani H . Erythromycin decreases the time and improves the quality of EGD in patients with acute upper GI bleeding. Gastroenterol Hepatol Bed Bench. (2013) 6:195–201. PMC4017518 24834272

[31] Carbonell N Pauwels A Serfaty L Boelle P-Y Becquemont L Poupon R . Erythromycin infusion prior to endoscopy for acute upper gastrointestinal bleeding: a randomized, controlled, double-blind trial. Am J Gastroenterol. (2006) 101:1211–5. doi: 10.1111/j.1572-0241.2006.00582.x 16771939

[32] Coffin B Pocard M Panis Y Riche F Lainé M-J Bitoun A . Erythromycin improves the quality of EGD in patients with acute upper GI bleeding: a randomized controlled study. Gastrointest Endosc. (2002) 56:174–9. doi: 10.1016/s0016-5107(02)70174-0 12145593

[33] Frossard JL Spahr L Queneau PE Giostra E Burckhardt B Ory G . Erythromycin intravenous bolus infusion in acute upper gastrointestinal bleeding: a randomized, controlled, double-blind trial. Gastroenterology. (2002) 123:17–23. doi: 10.1053/gast.2002.34230 12105828

[34] Na HK Jung H-Y Seo DW Lim H Ahn JY Lee JH . Erythromycin infusion prior to endoscopy for acute nonvariceal upper gastrointestinal bleeding: a pilot randomized controlled trial. Korean J Intern Med. (2017) 32:1002–9. doi: 10.3904/kjim.2016.117 28352063 PMC5668395

[35] Pateron D Vicaut E Debuc E Sahraoui K Carbonell N Bobbia X . Erythromycin infusion or gastric lavage for upper gastrointestinal bleeding: a multicenter randomized controlled trial. Ann Emerg Med. (2011) 57:582–9. doi: 10.1016/j.annemergmed.2011.01.001 21333385

[36] Altawil L Al Hayek M Nounou MV Dawwa A Barakat O Sawaf B . Comparative effectiveness of prokinetic agents with or without nasogastric lavage for improving endoscopic visualization in upper gastrointestinal bleeding: a systematic review and network meta-analysis. Dig Dis. (2026) 44(3):342–55. doi: 10.1159/000551800 41911096 PMC13258244

[37] Guyatt GH Oxman AD Vist GE Kunz R Falck-Ytter Y Alonso-Coello P . GRADE: an emerging consensus on rating quality of evidence and strength of recommendations. BMJ. (2008) 336:924–30. doi: 10.1136/bmj.39489.470347.ad 18436948 PMC2335261

