# Supplementary material for: Role of prokinetic drugs prior to endoscopy in acute upper gastrointestinal bleeding: a systematic review and meta-analysis
Source: Front Gastroenterol (Lausanne). 2026 Sep 16;5:1930145. doi: 10.3389/fgstr.2026.1930145 (PMC13623750; doi:10.3389/fgstr.2026.1930145)
Supplement: Supplementary file 2 [file Table2.docx]

# 1. Search and Screening

*Appendix Table A1. Detailed Search Strategy Across Databases*

| **Database** | **Search strategy** | **Records identified** |
| --- | --- | --- |
| PubMed / MEDLINE | (("Gastrointestinal Hemorrhage"[Mesh] OR "upper gastrointestinal bleeding"[tiab] OR "upper gastrointestinal hemorrhage"[tiab] OR "upper GI bleeding"[tiab] OR "upper GI hemorrhage"[tiab] OR UGIB[tiab] OR UGIH[tiab] OR hematemesis[tiab] OR haematemesis[tiab] OR melena[tiab] OR melaena[tiab] OR "variceal bleeding"[tiab] OR "variceal hemorrhage"[tiab] OR "nonvariceal upper gastrointestinal bleeding"[tiab] OR NVUGIB[tiab]) AND ("Erythromycin"[Mesh] OR erythromycin[tiab] OR "Metoclopramide"[Mesh] OR metoclopramide[tiab] OR prokinetic*[tiab] OR gastrokinetic*[tiab] OR "motilin agonist"[tiab] OR "motilin receptor agonist"[tiab]) AND ("Endoscopy, Gastrointestinal"[Mesh] OR endoscop*[tiab] OR "upper endoscopy"[tiab] OR esophagogastroduodenoscopy[tiab] OR oesophagogastroduodenoscopy[tiab] OR EGD[tiab] OR OGD[tiab]) AND (randomized controlled trial[pt] OR controlled clinical trial[pt] OR randomized[tiab] OR randomised[tiab] OR randomly[tiab] OR placebo[tiab] OR trial[tiab])) | 134 |
| Scopus | TITLE-ABS-KEY(("upper gastrointestinal bleeding" OR "upper gastrointestinal hemorrhage" OR "upper GI bleeding" OR "upper GI hemorrhage" OR UGIB OR UGIH OR hematemesis OR haematemesis OR melena OR melaena OR "variceal bleeding" OR "variceal hemorrhage" OR "nonvariceal upper gastrointestinal bleeding" OR NVUGIB) AND (erythromycin OR metoclopramide OR prokinetic* OR gastrokinetic* OR "motilin agonist" OR "motilin receptor agonist") AND (endoscop* OR "upper endoscopy" OR esophagogastroduodenoscopy OR oesophagogastroduodenoscopy OR EGD OR OGD) AND (random* OR placebo OR trial OR "controlled trial")) | 304 |
| Web of Science Core Collection | TS=(("upper gastrointestinal bleeding" OR "upper gastrointestinal hemorrhage" OR "upper GI bleeding" OR "upper GI hemorrhage" OR UGIB OR UGIH OR hematemesis OR haematemesis OR melena OR melaena OR "variceal bleeding" OR "variceal hemorrhage" OR "nonvariceal upper gastrointestinal bleeding" OR NVUGIB) AND (erythromycin OR metoclopramide OR prokinetic* OR gastrokinetic* OR "motilin agonist" OR "motilin receptor agonist") AND (endoscop* OR "upper endoscopy" OR esophagogastroduodenoscopy OR oesophagogastroduodenoscopy OR EGD OR OGD) AND (random* OR placebo OR trial OR "controlled trial")) | 56 |
| Cochrane Library / CENTRAL | (("upper gastrointestinal bleeding" OR "upper gastrointestinal hemorrhage" OR "upper GI bleeding" OR "upper GI hemorrhage" OR UGIB OR UGIH OR hematemesis OR haematemesis OR melena OR melaena OR "variceal bleeding" OR "variceal hemorrhage" OR "nonvariceal upper gastrointestinal bleeding" OR NVUGIB) AND (erythromycin OR metoclopramide OR prokinetic* OR gastrokinetic* OR "motilin agonist" OR "motilin receptor agonist") AND (endoscop* OR "upper endoscopy" OR esophagogastroduodenoscopy OR oesophagogastroduodenoscopy OR EGD OR OGD)) | 89 |
| Embase | ('upper gastrointestinal bleeding'/exp OR 'gastrointestinal hemorrhage'/exp OR 'upper gastrointestinal bleeding':ti,ab OR 'upper gastrointestinal hemorrhage':ti,ab OR 'upper GI bleeding':ti,ab OR 'upper GI hemorrhage':ti,ab OR UGIB:ti,ab OR UGIH:ti,ab OR hematemesis:ti,ab OR haematemesis:ti,ab OR melena:ti,ab OR melaena:ti,ab OR 'variceal bleeding':ti,ab OR 'variceal hemorrhage':ti,ab OR 'nonvariceal upper gastrointestinal bleeding':ti,ab OR NVUGIB:ti,ab) AND ('erythromycin'/exp OR erythromycin:ti,ab OR 'metoclopramide'/exp OR metoclopramide:ti,ab OR prokinetic*:ti,ab OR gastrokinetic*:ti,ab OR 'motilin agonist':ti,ab OR 'motilin receptor agonist':ti,ab) AND ('endoscopy'/exp OR endoscop*:ti,ab OR 'upper endoscopy':ti,ab OR esophagogastroduodenoscopy:ti,ab OR oesophagogastroduodenoscopy:ti,ab OR EGD:ti,ab OR OGD:ti,ab) AND ('randomized controlled trial'/exp OR random*:ti,ab OR placebo:ti,ab OR trial:ti,ab OR 'controlled trial':ti,ab) | 50 |

# 2. Risk of Bias Assessment

*Appendix Table A2. Detailed Quality Assessment: Risk of Bias Assessment Using Cochrane RoB 2. The risk of bias was assessed using the Cochrane Risk of Bias 2 tool for randomized trials. Judgments were made for the primary outcome of adequate gastric emptying or adequate endoscopic visualization at index endoscopy. Domains assessed were: D1, bias arising from the randomization process; D2, bias due to deviations from intended interventions; D3, bias due to missing outcome data; D4, bias in measurement of the outcome; and D5, bias in selection of the reported result. Overall judgments followed RoB 2 guidance and were categorized as low risk, some concerns, or high risk of bias. Abbreviations: D1–D5, RoB 2 bias domains; EGD, esophagogastroduodenoscopy; RoB 2, Cochrane Risk of Bias 2 tool.*

| **Study** | **D1: Randomization process** | **D2: Deviations from intended interventions** | **D3: Missing outcome data** | **D4: Outcome measurement** | **D5: Selection of reported result** | **Overall RoB 2 judgment** | **Main reason for judgment** |
| --- | --- | --- | --- | --- | --- | --- | --- |
| Ali Shah et al., 2020 | Low | Low | Low | Low | Some concerns | Some concerns | Computer-generated pharmacy randomization, identical placebo/erythromycin packages, and blinding of both patients and the endoscopist were reported. The endoscopist performing the procedure was blinded and assessed visualization using predefined subjective/objective criteria. However, the scoring system was modified and no prospective protocol or trial registration was identified; therefore, concerns remain regarding selection of the reported result. |
| Altraif et al., 2011 | Low | Low | Some concerns | Low | Some concerns | Some concerns | Computer-generated randomization, pharmacy-controlled allocation, identical treatment packages, and double blinding were reported. Both the endoscopist and patient were blinded to the study drug, supporting low risk for outcome measurement despite the subjective visualization endpoint. However, 102 patients were randomized/treated and only 90 patients with confirmed variceal bleeding were included in the final analysis, and a clearly prespecified publicly available analysis plan was not fully available. |
| Carbonell et al., 2006 | Low | Low | Low | Low | Some concerns | Some concerns | Computer randomization, pharmacy allocation, identical treatment packaging, and a randomized placebo-controlled double-blind design were reported. The primary visualization outcome was assessed using both subjective and objective criteria within this double-blind protocol; therefore, measurement bias was considered unlikely. However, one patient was excluded after randomization and no clearly prespecified publicly available protocol was identified. |
| Coffin et al., 2002 | Some concerns | Some concerns | Low | Low | Some concerns | Some concerns | Randomization was performed using a standard table, with unclear allocation concealment. The study was not double-blind because there was no placebo and the ICU team was not blinded. However, the endoscopists who performed the EGDs were blinded to treatment allocation, supporting low risk for outcome measurement of the subjective visualization endpoint. No clearly prespecified publicly available protocol was identified. |
| Ehsani Ardakani et al., 2013 | Some concerns | Some concerns | Low | Some concerns | Some concerns | Some concerns | The study was described as randomized and double-blind, but the method of sequence generation, allocation concealment, and blinding procedures were not fully described. Because the visualization outcome was partly subjective and the report did not clearly specify whether the endoscopist or visualization assessor was blinded to treatment allocation, Domain 4 was judged as some concerns. No clearly prespecified publicly available protocol was identified. |
| Frossard et al., 2002 | Low | Low | Low | Low | Low | Low | Double-blind placebo-controlled randomized design was reported, with pharmacy allocation, identical syringes, and treatment identity withheld until final analysis. The primary outcome included subjective and objective visualization assessment, but the double-blind design and identical placebo/intervention preparation supported low risk for outcome measurement. The study used intention-to-treat analysis and had no major concerns across RoB 2 domains. |
| Manupeeraphant et al., 2024 | Some concerns | Low | Some concerns | Low | Some concerns | Some concerns | This was a multicenter double-blind randomized controlled trial. Treatments were delivered in a double-blinded manner, and both endoscopists and participants were blinded during the procedure, supporting low risk for outcome measurement. However, trial registration occurred after enrollment had started, and the final analysis excluded participants with incomplete protocol or refusal to stay after endoscopy, leading to some concerns for randomization/reporting and missing outcome data. |
| Na et al., 2017 | Some concerns | Low | Low | Low | Some concerns | Some concerns | Stratified randomization was reported, and the endoscopist who evaluated visualization during EGD was unaware of group assignment, supporting low risk for outcome measurement. However, allocation concealment was not clearly described, and no prospectively registered analysis plan was identified; therefore, there were some concerns for the randomization process and selection of reported results. |
| Pateron et al., 2011 | Low | Low | Low | Low | Low | Low | Central interactive voice-response randomization, multicenter design, and intention-to-treat analysis were reported. Endoscopists were unaware of treatment assignment, and measures were taken to prevent disclosure of allocation before the patient arrived in the endoscopy room. Missing primary outcome data were treated conservatively as failures. Overall, the trial was judged to be at low risk of bias. |
| Vimonsuntirungsri et al., 2024 | Low | Low | Some concerns | Low | Low | Some concerns | Concealed computer-generated block randomization and blinding were appropriate. Patients and endoscopists were blinded to treatment allocation, and reference endoscopic photographs were internally validated by another experienced endoscopist blinded to randomization allocation, supporting low risk for outcome measurement. However, 6 of 68 randomized participants were excluded before the primary analysis because of protocol violations, leading to some concerns for missing outcome data and the overall judgment. |

*Supplementary Table A3 summarizes the study-level safety reporting. Overall, available trial data did not identify a consistent signal of serious harm, but adverse-event definitions, monitoring intensity, and reporting completeness varied substantially across trials. Therefore, safety conclusions remain limited and should be interpreted separately from efficacy outcomes.*

| **Study** | **Agent/intervention** | **Safety/adverse-event reporting in trial** | **Reported adverse events or complications** | **QT prolongation / arrhythmia** | **Extrapyramidal symptoms** | **Aspiration / respiratory complications** | **Interpretation** |
| --- | --- | --- | --- | --- | --- | --- | --- |
| **Ali Shah et al., 2020** | Oral erythromycin vs placebo | Endoscopy-related complications were listed as secondary outcomes. | No significant side effects or endoscopy-related complications were reported in either group; no deaths occurred during the study period. | Not specifically reported. | Not applicable. | Not specifically reported. | No safety signal reported, but detailed adverse-event categories were limited. |
| **Altraif et al., 2011** | IV erythromycin vs placebo | Drug-related adverse events and aspiration pneumonia were reported. | No adverse events were observed with erythromycin. | No drug-related adverse effects were reported; specific QT data were not reported. | Not applicable. | Aspiration pneumonia was numerically higher in placebo than erythromycin, 4.7% vs 0.0%, not statistically significant. | No erythromycin-related safety signal reported. |
| **Carbonell et al., 2006** | Gastric lavage + IV erythromycin vs gastric lavage + placebo | Adverse effects were actively recorded during and after infusion, including nausea, vomiting, diarrhea, gastric contractions, and liver enzymes. | No adverse event was observed in either group. Gastric contractions reducing endoscopy quality occurred in 20% with erythromycin vs 14% with placebo. | QT prolongation was an exclusion criterion, but QT events during follow-up were not separately reported. | Not applicable. | Not specifically reported as an event. | No major adverse events reported; gastric contractions were noted. |
| **Coffin et al., 2002** | IV erythromycin vs no treatment | Adverse events were recorded. | No adverse event was observed; no erythromycin-related adverse event was reported. | Not specifically reported. | Not applicable. | Not specifically reported. | No erythromycin-related safety signal reported. |
| **Ehsani Ardakani et al., 2013** | Nasogastric tube/lavage + IV erythromycin vs nasogastric tube/lavage + placebo | Adverse events were recorded. | No complication was reported throughout the study. | Not specifically reported. | Not applicable. | Not specifically reported. | No complications reported, but event categories were not detailed. |
| **Frossard et al., 2002** | IV erythromycin vs placebo | Treatment safety was assessed by monitoring adverse effects after infusion. | Erythromycin group: 3 urgent bowel movements and 1 moderate abdominal cramps. Placebo group: 1 headache 30 minutes after infusion. | Not specifically reported. | Not applicable. | No complications were noted in the abstracted safety summary. | Mild transient symptoms were reported; no serious safety signal. |
| **Manupeeraphant et al., 2024** | IV metoclopramide vs placebo | Adverse events were recorded, and an endoscopic team monitored participants during the procedure. | After a single dose of metoclopramide, no adverse event was observed. | Not specifically reported. | Not specifically reported as occurring; the discussion notes that no documented adverse effects occurred after single-dose metoclopramide. | Two participants in the placebo group had post-endoscopy aspiration pneumonia. | No metoclopramide-related safety signal reported. |
| **Na et al., 2017** | IV erythromycin vs gastric lavage vs erythromycin + gastric lavage | Complications related to erythromycin infusion or gastric lavage were secondary outcomes. | No complications were reported. | Not specifically reported. | Not applicable. | Not specifically reported. | No complications associated with erythromycin infusion or gastric lavage. |
| **Pateron et al., 2011** | IV erythromycin vs nasogastric lavage vs erythromycin + lavage | Adverse effects related to erythromycin infusion or nasogastric tube placement were recorded; pain after nasogastric tube placement was assessed by visual analog scale. | No adverse effects were reported in any group. Nasogastric tube placement was associated with pain, and about one-quarter experienced severe pain. | Not specifically reported. | Not applicable. | Not specifically reported. | No severe adverse effects reported; lavage/tube discomfort was clinically relevant. |
| **Vimonsuntirungsri et al., 2024** | IV metoclopramide vs placebo | Serious adverse events and specific metoclopramide-related events were monitored. | No complications or serious adverse events were reported, including anaphylaxis, extrapyramidal reaction, or cardiac arrhythmia. | No cardiac arrhythmia reported. | No extrapyramidal reaction reported. | Not specifically reported. | Most detailed metoclopramide safety reporting; no serious safety signal identified. |
